# Advanced deep learning modeling to enhance detection of defective photovoltaic cells in electroluminescence images

**DOI:** 10.1038/s41598-025-14478-y

**Published:** 2025-08-27

**Authors:** Mostafa A. Ebied, Amr Munshi, Shakir A. Alhuzali, Mohamed M. El-sotouhy, Amr I. Shehta, M. S. Elborlsy

**Affiliations:** 1 Electronics Technology Department, Faculty of Technology and Education, Beni-Suef University, Banī Suwayf, Egypt; 2 Department of Computer and Network Engineering, College of Computing, Umm Al-Qura University, Makkah, Saudi Arabia; 3 College of Computing, Umm Al-Qura University, Makkah, Saudi Arabia; 4 Power Electronics and Energy Conversion Department, Electronics Research Institute (ERI), Cairo, Egypt; 5 Department of Information System, Faculty of Computers and Artificial Intelligence, Helwan University, Cairo, Egypt; 6 Process Control Technology Department, Faculty of Technology and Education, Beni-Suef University, Beni-Suef, Egypt

**Keywords:** Deep learning, Defect classification, Regression analysis, Generative adversarial network, Electroluminescence imaging, Photovoltaic (PV), Energy harvesting, Energy infrastructure, Energy storage, Nuclear energy, Renewable energy

## Abstract

This paper discusses a deep learning approach for detecting defects in photovoltaic (PV) modules using electroluminescence (EL) images. The method addresses key challenges in two practical areas: Creating high-quality EL images to overcome imbalance issues in existing datasets. This is accomplished by employing generative adversarial network (GAN) properties to generate new images. Enhancing training efficiency and performance through a one-cycle policy with optimized learning rate settings, designed to overcome hardware limitations. The research highlights that while automatic defect classification in PV modules is gaining attention as an alternative to visual/manual inspection, the process remains challenging due to the inhomogeneous nature of cell cracks and complex backgrounds in crystalline solar cells. A comparison was made between popular deep learning models (Densenet169, Densenet201, Resnet101, Resnet152, Senet154, Vgg16, and Vgg19) to assess the effectiveness of our approaches on multiple variants of our dataset. We also observe a shift in the phenomenon of moving the threshold in regression estimates because of employing a policy that uses a dynamic threshold instead of a standard threshold (0.5). We have employed two different categorizations that use binary numbers; the first employs four classes (0%, 33%, 67%, and 100%), while the second employs eight classes that are identical to four classes. However, each class has two varieties (monocrystalline and polycrystalline) and a boundary beyond which results will be obtained. Based on the performance results, it was found that the pre-trained Resnet152 model achieved the highest classification accuracy (90.13% for Datasets) of all approaches. Additionally, we have demonstrated that approaches that utilize over-sampling have the greatest performance. These findings emphasize the strength and innovation of our approach, combining advanced data augmentation, adaptive thresholding, and optimized learning strategies. The proposed system not only achieved a peak classification accuracy of 90.13% using ResNet152 but also demonstrated high robustness, reduced training time, and superior generalization across defect types and cell categories. This positions our framework as a scalable and deployment-ready solution for real-world photovoltaic quality inspection systems.

## Introduction

In recent years, global energy consumption has risen substantially and is projected to continue an upward trend. Meanwhile, conventional energy sources such as coal, oil, and natural gas are being depleted at an accelerating rate, leading to higher costs and raising both environmental and economic concerns. Given this growing demand, it is essential to explore alternative energy sources such as geothermal, solar, tidal, and wind power to ensure a sustainable and dependable energy future^1–3^. Among these, renewable energy technologies like photovoltaic (PV) systems play a vital role. In recent years, PV technology has seen notable advancements. Typically, PV panels are encased in an aluminum frame and covered with laminated glass to safeguard against various risks^4,5^. Despite these protective designs, PV modules are still vulnerable to thermal and mechanical damage, particularly during manufacturing, transportation, and installation stages^6,7^. After installation, they are further exposed to environmental factors such as rain, snow, wind, and lightning, which can cause cracks and additional cell-level defects^8,9^. These issues compromise the integrity of the modules, leading to greater energy losses, reduced conversion efficiency, and a decline in overall system performance^10,11^. Evaluating the solar modules’ quality can be a challenging task, even for experts who have received training in visual identification. While some defects, such as visible cracks in the glass, are easy to spot, many other defects can negatively impact the efficiency of PV modules but are hidden from view by the naked eye. On the other hand, not all visible defects necessarily lead to a decrease in the efficiency of the module. To accurately assess the efficiency of a photovoltaic (PV) module, its electrical output must be measured. However, this process typically requires manual diagnostics and interaction with individual units, making it inefficient and impractical for large-scale solar installations comprising thousands of modules. Additionally, such measurements capture only a single moment in time and may fail to detect minor cracks or defects that can gradually expand and impair performance over time^12^. As a more practical alternative, infrared (IR) imaging provides a non-invasive, contactless method for evaluating the condition and quality of solar modules.

Even experienced professionals may struggle to evaluate solar module quality effectively, since numerous imperfections remain undetectable through visual inspection alone. While some issues, such as cracks in the glass, are easily noticeable, other flaws that significantly impact the efficiency of a photovoltaic (PV) module may go undetected through visual inspection alone. Conversely, not all visible imperfections necessarily lead to performance degradation. Accurate evaluation of a module’s efficiency typically requires electrical output measurements. However, this process involves manual testing of individual modules, making it impractical for large-scale solar farms containing hundreds or thousands of units. While conventional testing methods provide limited temporal snapshots that may miss evolving defects, infrared imaging technology delivers a more robust solution for contactless PV module quality assessment and performance monitoring.

Photovoltaic module damage frequently manifests as solar cells that become partially or completely disconnected from the circuit. When this occurs, the affected cells cease energy conversion and begin accumulating heat, causing them to emit detectable infrared radiation. While IR cameras can capture this thermal signature, the technique’s limited resolution often prevents identification of minor defects like microcracks that haven’t yet affected performance. For more precise fault detection, electroluminescence (EL) imaging serves as a complementary diagnostic method^13,14^. EL imaging provides superior resolution to IR techniques, enabling identification of subtle structural abnormalities and incipient defects that might otherwise go undetected.

In electroluminescence (EL) imaging, defective solar cells exhibit reduced luminescence intensity as disconnected regions cannot emit radiation. The EL imaging process involves applying an external current to the photovoltaic module, inducing light emission at ~ 1150 nm wavelength. This radiation is detected by a silicon-based CCD sensor. Unlike thermal imaging where heat diffusion causes blurring, EL provides superior spatial resolution for identifying microcracks and fine defects. However, the technique’s practical application is constrained by the need for expensive specialized equipment, time-consuming procedures, and expert interpretation. Our proposed solution overcomes these challenges through an automated EL image classification system that minimizes manual intervention and reduces dependency on expert analysis.

**Fig. 1:**
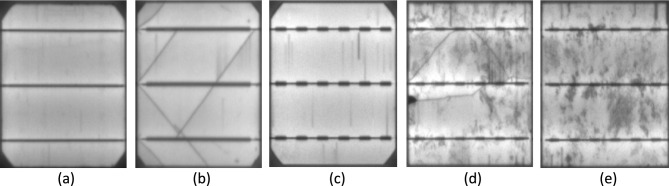
Application of a GAN-based augmentation method to generate EL images of (**a**) a defect-free cell; (**b**) a microcrack in monocrystalline silicon; (**c**) a finger-interruption in monocrystalline silicon; (**d**) a microcrack in polycrystalline silicon; and (**e**) a finger-interruption in polycrystalline silicon.

There are two main categories of defects found in solar modules^15^. The first category includes intrinsic deficiencies that result from material properties, like crystal grain limitations and dislocations. The second category consists of process-induced extrinsic defects, as microcracks and disruptions, that gradually decrease the total efficiency of the module. Figure 1 provides an illustrative case of different defect types in both polycrystalline and monocrystalline cells. Specifically, Figs. 1(a) and Figs. 1(b) showcase universal material defects that arise during the production method, like finger interruptions. However, these defects do not essentially impact the lifetime of the affected solar panel except when they are produced by excessive strain at the joints. The impact of finger interruptions on efficiency is a multifaceted phenomenon influenced by factors such as position, size, and the number of interruptions^16,17^. Figure 1(c), Figs. 1(d), and Figs. 1(e) visually depict common issues associated with finger interruptions, including deteriorating cell interconnections, microcracks, and electrically isolated or deteriorated cell components, all of which are known to diminish module efficiency. Detecting microcracks, in particular, necessitates the use of high-resolution cameras.

Defection during monitoring can be aimed at different targets. Identification of defects at the precise location within a solar module enables subsequent monitoring of those damaged regions in high resolution. In contrast, the specific defect position in the solar cell becomes a minor consideration when assessing the quality of a complete PV module. It is more important for this purpose to assess the total likelihood of a defect, which then allows one to find out promptly and potentially related to later efficiency loss in a PV module. Deep learning is considered an effective tool in identifying and classifying patterns, and it has found its way into applications such as autopilot, robotic automation, and healthcare, among others. Numerous deep learning-based techniques, including Alex Net^18^, VGG^19^, Google Net^20^, and Res Net^7,21^, have been developed for automatic categorization in literature. The effectiveness of these approaches in accurately classifying defects in PV modules heavily depends on the process of training, which requires a satisfactory sum of image samples. This offers an important challenge when it comes to implementing defect analysis and detection using deep learning. However, a promising solution has emerged in the form of GANs^22^. GANs can generate novel images by extracting properties from the input image dataset, offering a potential solution to this problem. Various GAN-based models, like DCGAN^23^, WGAN^24,25^, and WGAN-GP^26^, along with other variants, have been developed. Previous research and solutions have demonstrated the viability of automatically detecting and analyzing PV modules using deep learning^27^. In conclusion, it is evident that the current solutions have their limitations, and there is still a need for extensive research and exploration to achieve effectual defect discovery and analysis of PV modules using EL images. To address the following objectives: (1) enhancing data augmentation techniques, (2) expanding the scope of module defect analysis, and (3) improving the robustness and detection accuracy of machine learning models, this study explores an automated PV module flaw-detecting system utilizing EL images.

The core technical advancements of this study can be delineated across three primary domains:

To optimize performance and reduce training time in deep learning models, a CNN is paired with an effective learning rate scheduler known as the one-cycle policy. This approach enhances both the performance and efficiency of the training stage, which is crucial in any deep learning model. During model training, the one-cycle learning rate policy was used to accelerate convergence and improve generalization. This scheduler adjusts the learning rate in a triangular pattern, starting with a linear increase followed by a gradual decrease. Compared to other common scheduling methods, such as step decay and cosine annealing, the one-cycle approach yielded faster convergence and slightly higher validation accuracy in our experiments, particularly when using deep CNNs on the augmented EL dataset. It is crucial to consider the phenomenon of a shifting threshold in regression prediction after the training stage. This indicates the presence of a biased prediction that needs to be accounted for before applying a standard threshold, as the optimal threshold point for each model is dynamic and subject to change. This study conducts comprehensive evaluation and validation of prominent deep learning architectures by benchmarking their performance against established reference models, including DenseNet169, DenseNet201, ResNet101, ResNet152, SENet154, VGG16, and VGG19. We perform extensive comparative experiments to assess model performance across varying hyperparameters and training configurations.

This study presents several important contributions that distinguish it from previous works. First, it introduces the use of generative adversarial networks to generate high-quality electroluminescence images, which effectively addresses the issue of dataset imbalance and enriches the diversity of defect representations. Second, the research proposes a dynamic thresholding technique that enhances the accuracy of regression-based classification by adapting to model bias and improving decision boundaries. Third, the adoption of a one-cycle learning rate policy significantly improves training efficiency and model generalization, reducing computation time while maintaining high performance. Additionally, the study develops a dual-level classification framework that combines defect severity with material type, offering a more detailed and realistic analysis of photovoltaic cell conditions. These contributions collectively form an innovative and integrated approach that advances the current state of automated defect detection in photovoltaic modules using deep learning and electroluminescence imaging.

This paper is structured as follows: Section "Linked works" provides a comprehensive review of relevant literature, establishing the theoretical foundation for the study. Section "Materials and Methods" describes the experimental methodology and materials employed in the research. Section "Experimental results" presents and analyzes the experimental results, discussing their significance and practical implications. Finally, Sect. 5 concludes the study by synthesizing key findings and proposing valuable avenues for future investigation.

## Linked works

Visual assessment of solar modules using EL photography is an area of ongoing study. Many of the associated studies, conversely, concentrate on detecting specific inherent or extrinsic flaws, but then again, they don’t have a way of predicting the ultimate effect of these flaws on the efficiency of solar panels. The discovery of surface irregularities of the EL images of solar cells is associated with health monitoring. However, it’s crucial to recognize that specific errors in solar cells are observed only via EL photodiodes in the context of modules that are composed of PV cells. For example, solar cells that are completely isolated appear as dark areas with a similar appearance to Fig. 1(d)) and, therefore, lack the equivalent of structural flaws. Moreover, the irregularities on the surface of the solar wafer (such as finger pauses) are frequently misinterpreted as cell cracks, although they have no significant effect on the loss of power. In terms of visual assessment of solar panels^28^ the authors utilize the Fourier transform to reconstruct images of polycrystalline PV modules to identify cells that are defective in their EL properties. The intended extrinsic flaws are (pequenos) flecks of water, glass, or plastic that are intended to be broken or cracked. Fourier image reconstruction is employed for eliminating potential flaws by setting the high-frequency coefficients that are related to the track and bar relics to zero. The demonstration of the spectrum then is converted vertebral into the spatial field. Flaws can be detected by analyzing the intensity differences between the original and high-pass filtered images. However, this technique struggles to identify defects with complex geometries due to its inherent form-based assumptions^29,30^. Supervised learning approaches using Independent Component Analysis (ICA) have been proposed for defect detection, where defect-free solar cell images are used to derive ICA basis images. While achieving 93.40% accuracy with a small training set of 300 sub-images, this method cannot distinguish between material imperfections (e.g., finger interruptions) and actual cell failures. Moreover, it detects surface irregularities but cannot predict future power loss^29,30^. For polycrystalline solar cells^31,32^ developed a microcrack detection method combining anisotropic diffusion with shape analysis. Although effective for microcracks, this approach fails to identify other defects like electrically isolated cells that appear dark in EL images. Similarly^33^, proposed an automated method for detecting finger interruptions in monocrystalline cells using binary clustering of candidate regions. However, finger interruptions alone are unreliable predictors of future performance degradation. Recent advances in optical inspection have seen deep learning surpass traditional pattern recognition methods. Notably, while CNNs have been applied to various inspection tasks, no architecture has been specifically designed for EL image analysis. Relevant work includes^34^:’s system for predicting power loss, defect location, and contamination type from RGB images^35^‘s end-to-end max-pooling CNN for steel defect classification, which outperformed SVM classifiers by at least 2 × despite limited data (2,281 training and 646 test images)^36^ employed a similar approach for concrete crack detection across various environmental and lighting conditions^37^. applied deep learning to structural health monitoring in aerial imagery, while^38^ explored defect localization using advanced learning-based segmentation for region recommendation with a real-time Faster R-CNN framework^39^. utilized semantic segmentation for concrete fracture detection. In medicine, deep neural networks have classified various skin cancers^40^, achieving high accuracy through end-to-end training on 129,450 clinical images across 2032 diseases^41^. presented PV module defect verification using IR imaging and module optimizer control, classifying substring faults from open/short circuits, bypass diode issues, cell faults, and undefined optimizer problems with 82.9% accuracy^42^. developed a deep convolutional neural network for automated damaged solar cell detection with 0.76% sensitivity.

Ref^43^. This research demonstrates an automated system for detecting solar module defects in thermal imagery using isolated deep learning and enhanced model transfer techniques, achieving exceptional accuracy rates of 98.67% and 99.23% respectively. In related work^44^, developed a system for defect detection and classification in South African solar installations using both traditional machine learning (SVM with polynomial and RBF kernels) and deep learning approaches (VGG-16 and MobileNet architectures). Their results showed 89.5% accuracy for CNN-based methods and 91.2% for feature-based detection approaches^45^. implemented solar cell surface defect inspection using multispectral CNNs, reaching 94.30% accuracy^46^. extracted degradation features from cyclic EL images during accelerated exposure tests and classified them using both supervised methods (achieving over 98% accuracy for five features) and unsupervised clustering (yielding two clusters with 66% consistency)^11^. compared CNN and SVM approaches for solar cell defect classification, with CNN achieving 91.58% accuracy, outperforming feature extraction-based SVM methods (HOG: 69.95%, KAZE: 71.04%, SIFT: 68.90%, SURF: 72.74%)^47^. developed the EL Eval-2 algorithm for automated solar cell categorization using EL photography, which outperformed trained manufacturer personnel in quality assessment^48^. created a novel optical CNN design for solar cell defect detection in EL images, achieving 93.02% accuracy^49^. developed a fully automatic segmentation method using the ELEval-2 algorithm, achieving a 94.47% median weighted Jaccard index and 97.54% F1 score^50^. presented generalized mechanistic PV module performance prediction using CNNs with 95% accuracy^51^. extended EL image datasets using GANs with their AC-PG GAN model, improving CNN classification accuracy by up to 14%^52^. developed a technique for microcrack detection in solar cells using binary and discrete Fourier transform image processing^28^. implemented automatic defect identification for solar cell modules using CNN-based deep learning, achieving 83% accuracy^19^. The study introduced an automated classification system for defective solar cells in electroluminescence (EL) images, employing a hybrid approach combining Support Vector Machines (SVMs) and Convolutional Neural Networks (CNNs). The proposed framework achieved classification accuracies of 88.42% using SVM and 82.9% with CNN architectures^53^. proposes a deep-learning method for PV cell defect detection using EL images. It addresses data scarcity and imbalance via data enhancement and category weighting and improves feature extraction with a ResNet152–Xception fusion and coordinate attention. The model achieves 96.17% accuracy in binary classification and 92.13% in multiclass tasks, outperforming several CNN benchmarks^54^. presents a deep learning pipeline for detecting, locating, and segmenting PV cell defects in EL images. It combines object detection (Faster-RCNN), classification (EfficientNet), and weakly supervised segmentation (autoencoder) to improve accuracy. The modular design allows for future upgrades and new functionalities^55^. proposes a cost-efficient deep learning method for automated PV cell defect segmentation in EL images, reducing annotation costs by 60% while adapting to new cell types^56^. Automated CNN-based defect detection in PV cells using EL images achieves 99.8% accuracy, with pseudo-coloring for enhanced visualization and fast segmentation^57^. DCGAN generates 10,000 synthetic PV cell EL images to enhance ML defect detection, validated by high Inception Score (2.3) and low FID (15.8)^58^. LumiNet, a CNN framework, enables efficient solar cell binning and defective detection from EL images, matching IV tester accuracy while overcoming its cost/speed limitations^59^. Hybrid DL system (Inception-V3 + ResNet50) automates PV defect detection in EL images with 98.15% binary and 95.35% multi-class accuracy. Table 1 summarizes the critical components of related studies, including their methodologies, datasets, performance metrics, and identified limitations.

**Table 1:** Comprehensive analysis of the literature study.

Ref	Methodology	Dataset	Performance matrices	Limitations
^28^	• Replaces weight clipping with gradient penalty. Enforces Lipschitz constraint on critic. Samples interpolated points between real and fake data. Uses two-sided penalty for gradient norms. Omits batch normalization in critic	• Toy datasets (Swiss Roll) • CIFAR-10 • LSUN Bedrooms • Google Billion Word • MNIST subset	• Inception Score (SOTA on CIFAR-10) • Training stability (200 + architectures) • Faster convergence than WGAN • Qualitative text generation	• Higher computational cost • Sensitive to penalty coefficient • Struggles with discrete data • Critic overfits small datasets • No batch normalization in the critic
^29^	• The paper proposes a deep learning-based method for defect detection in PV modules using EL images • It combines GAN for data augmentation and CNN for defect classification	• The dataset includes 1800 EL images (450 per defect type: micro-crack, finger-interruption, break, defect-free) • Samples are augmented using GAN and traditional image processing	• Accuracy is the main metric. The proposed CNN model achieves 83% accuracy on validation data, outperforming VGG16, ResNet50, InceptionV3, and MobileNet	• The model struggles with multiple coexisting defects • Overfitting occurs with excessive epochs • Performance depends on high-quality EL images, which are limited
^30^	• Fourier-based self-reference method detects solar cell defects (cracks, finger interruptions) in EL images by removing defect frequencies, reconstructing a defect-free image, and comparing it with the original. A Hough transform detects defect angles in the spectrum	• The study uses EL images of multicrystalline solar cells, each 550 × 550 pixels • The dataset includes defect-free samples and defective samples with cracks, breaks, and finger interruptions	• The method correctly identified all 15 defective samples and had no false alarms in 308 defect-free samples • Processing time was 0.29 s per image	• The method assumes defects are line- or bar-shaped. It may misclassify grain boundaries with similar orientations as defects • Parameter tuning (e.g., band-rejection width) is needed for different image sizes or resolutions
^31^	• Solar cell defect detection using ICA: train basis images on defect-free data, then detect defects via reconstruction errors	• The dataset includes EL images of multicrystalline solar modules. Each module has 36 solar cells (208 × 208 pixels) • The test set has 80 samples (28 defect-free, 52 defective)	• Recognition rate (R%) • Mean recognition rate: 93.4% for image reconstruction	• No defect shape/location details • Training-dependent performance • Morphological smoothing has minor impact • Only detects defects, not classifies types
^32^	• Used enhanced anisotropic diffusion with sigmoid-based adaptive thresholding • Segmentation via double thresholding & intensity tracing • Shape feature extraction using Angular Radial Transform (ART) • SVM-based defect classification	• 600 electroluminescence (EL) images: 313 intact and 287 defective solar cells • Images were 8-bit grayscale, sized 1,178 × 1,178 pixels	• Sensitivity: 97% • Specificity: 80% • Accuracy: 88% • F-measure: 0.0821 for segmentation	• Minimum detectable crack: 6.22 mm (may miss finer defects) • Processing time: ~ 4.1 s/image (not real-time for high-throughput) • Sensitivity to irregular/noisy backgrounds (may reduce accuracy)
^33^	• Spectral clustering for finger interruption detection in solar cell EL images • Gray-level feature extraction from fingers • Training: Clusters feature into interrupted/non-interrupted classes • Uses nearest centroid classification for defect detection	• The experiments used 60 Mult crystalline solar cells with various defects • Each cell had 72 or 82 fingers per ROI • EL images were 1024 × 1024 pixels with 12-bit gray levels • The dataset included intrinsic and extrinsic defects	• Accuracy, miss rate, and false alarm rate were calculated • Accuracy rates were 99.07% for top/bottom ROIs and 99.58% for middle ROIs • Miss rates were 6.89% and 2.39%, respectively • False alarm rates were 0.66% and 0.34%	• The method may miss interruptions if the defect ratio is below a threshold • It can misclassify noise resembling interrupted fingers • Performance varies slightly between ROIs due to height differences • The method relies on predefined parameters and training data
^34^	• Uses CNN-based DeepSolarEye for solar panel soiling analysis • Predicts power loss, soiling localization, and type from RGB images • Four-stage weakly supervised training avoids manual localization labels • Introduces BiDIAF block to improve localization	• PV-Net dataset with 45,754 solar panel images • Includes power loss labels, solar irradiance, and timestamps • Collected using two panels: one soiled, one reference	• Classification accuracy: 83.32% • Localization Jaccard Index: 66% • WebNN soiling type accuracy: 96.24% (dataset), 87% (web images) • BiDIAF improves classification by 3%, localization by 4%	• Requires power loss labels; no manual localization labels • Performance depends on environmental factors • Limited to RGB images; no IR or thermal data • Generalizability to unseen soiling types needs validation
^35^	• Used Max-Pooling Convolutional Neural Networks (MPCNN) for steel defect classification • Trained two architectures (5HL and 7HL) with stochastic gradient descent • Applied random translations (± 15%) for better generalization • Compared results to SVM and MLP classifiers using standard features (LBP, HOG, etc.)	• 7 defect classes from a real steel production line • 2281 training and 646 test images • Images resized to 150 × 150 pixels, preserving aspect ratio • Included intra-class variability and potential false positives	• Best MPCNN achieved 7% error rate. Outperformed standard feature-based classifiers (best: PHOG at 15.48%) • Committees of classifiers reduced error further but still underperformed MPCNN • Confusion matrices showed high accuracy for most classes	• Relied on pre-segmented defects; ignored detection errors • Limited by dataset size and variability • Training required GPU acceleration for efficiency • Hand-crafted features still outperformed MPCNN on one defect class
^36^	• CNN-based crack detection in concrete surfaces • Architecture: Convolution, pooling, ReLU, dropout, batch norm, softmax • Sliding window for large-image processing • Training: SGD with tuned hyperparameters (learning rate, momentum)	• The dataset consists of 332 images (277 for training/validation, 55 for testing) • Images are cropped into 40,000 smaller 256 × 256 pixel patches. The dataset includes varied conditions like lighting changes, shadows, and thin cracks • Images are manually labeled as“crack”or"intact."	• The CNN achieves 98.22% training accuracy and 97.95% validation accuracy • Testing on 55 new images yields 97% accuracy • Comparative studies show the CNN outperforms traditional methods (Canny and Sobel edge detection) in robustness and adaptability	• The method requires a large dataset for training. It cannot detect internal defects (e.g., crack depth) as it relies on surface images • Performance may degrade for rare defects with insufficient training data • Computational cost is high without GPUs
^37^	• Uses ultrasonic beacons (UBS) for navigation • Deep CNN for concrete crack detection • Geo-tagging for damage localization • Custom UAVs (Pixhawk 2.1 + Bebop2) with action cams & UBS	• Training: 40,000 images of cracked/intact concrete (256 × 256 pixels) • Test data: Video footage from UAV flights in indoor environments (classrooms E2-229, E2-399)	• Accuracy: 96.6% • Sensitivity: 91.9% • Specificity: 97.9%	• Manual flight tuning • 15-min flight endurance • UBS limited to 30 × 30 m (non-penetrating) • Offline CNN processing (6 s/frame)
^38^	• The paper proposes a Faster R-CNN-based method for detecting multiple structural damages • It modifies the Faster R-CNN architecture to classify five damage types • The method uses shared CNN layers between RPN and Fast R-CNN for feature extraction	• The dataset includes 2,366 images (500 × 375 pixels) labeled for five damage types: concrete cracks, steel corrosion (medium/high), bolt corrosion, and steel delamination • Images were collected from bridges and buildings • Data augmentation (horizontal flipping) was applied to the training set	• Average Precision (AP) was used for evaluation • The method achieved APs of 90.6% (concrete cracks), 83.4% (medium steel corrosion), 82.1% (high steel corrosion), 98.1% (bolt corrosion), and 84.7% (steel delamination), with a mean AP of 87.8%	• Minor errors occurred due to lighting conditions, camera angles, or small training datasets • Some misclassifications happened for corroded bolts and small cracks • The method requires a fixed camera distance (1.0–1.5 m) for optimal performance • Future work includes expanding the dataset and using UAVs for better angles
^39^	• Proposed a crack detection network (CSN) using deep learning-based semantic segmentation • Used a 2D Gaussian kernel and Brownian motion to generate synthetic crack images • Pre-trained CSN on MS-COCO dataset, then fine-tuned on crack datasets	• Collected 242 real crack images (plain and cluttered) • Created two integrated datasets: 100 and 200 synthetic cracks added to real data • Split data: 207 for training, 35 for validation	• Precision, recall, and accuracy are used for evaluation • CSN outperformed patch-based CNN in robustness and speed (0.2991 s vs. 578.38 s per image) • Tested under brightness, hue, and noise variations; CSN showed consistent performance	• Performance declined in extreme noise or low brightness • Struggled with cracks covered by leaves or debris • Synthetic cracks may not fully replicate real-world complexity • Limited dataset size (242 real images)
^40^	• Used a deep convolutional neural network (CNN) based on GoogleNet Inception v3 • Pretrained on ImageNet (1.28 million images) and fine-tuned on a dermatology dataset • Trained end-to-end using only pixels and disease labels	• 129,450 clinical images of 2,032 skin diseases • Included 3,374 dermoscopy images • Data from 18 open-access repositories and Stanford University Medical Center • Test sets comprised biopsy-proven images	• Matched performance of 21 board-certified dermatologists • Achieved AUC > 91% for keratinocyte carcinoma and melanoma classification • Sensitivity and specificity on par with experts • Validated using nine-fold cross-validation	• Rely on visual data only, excluding contextual clinical factors • Performance in real-world clinical settings is not fully validated • Limited by data availability for rare conditions • Potential biases in dermatologist-labeled training data
^41^	• Used drone-mounted IR cameras to inspect PV plants • Analyzed module optimizers’ electrical data • Compared IR findings with monitoring data	• 10 residential PV plants • Plants had 16.5–99.75 kWh capacity • Modules operated for 1–6 years	• Defect detection accuracy • Power and yield reduction • Temperature differences in IR images	• IR imaging provides only snapshots • Monitoring data lacks detailed defect causes • Edge cooling effects are hard to quantify
^42^	• Used a VGG-16 DCNN for detecting damaged PV cells • Trained on thermal images from drones • Applied data augmentation (flipping, rotating) to balance the dataset	• 3,336 thermal images (811 damaged, 2,525 normal) • Collected via drone with a FLIR Tau 2 camera • Manually labeled ground truth	• accuracy, precision, recall, F1-score. Balanced dataset improved results (e.g., F1-score: 0.69) • Data augmentation boosted performance (F1-score: 0.75 for rotated images)	• An unbalanced dataset initially reduced accuracy. Small dataset size • Limited to thermal images. Manual labeling is prone to human error
^43^	• Used isolated deep learning (trained from scratch) and developed a model transfer deep learning • Light CNN architecture with four convolutional layers, ReLU activation, L2 regularization, max pooling, batch normalization, and dropout	• 893 IR images of PV modules (normal and defective) • Defects included failed interconnections, cracks, resistive soldering, and shunting • Collected from lab experiments and online sources	• Isolated model: 98.67% accuracy (fivefold avg.) • Transfer model: 99.23% accuracy (fivefold avg.) • Real-time prediction speed (~ 13 ms per image) • High precision, recall, and F1 scores for both models	• Small dataset size may limit generalization • Misclassification of images with high current density or local shunts • Overfitting is observed with pre-trained models like VGG-16 • Manual labeling is required for defect categories
^44^	• Used thermal infrared imaging to detect and classify PV module defects • Applied SIFT descriptors with a bag of visual words model and random forest/SVM classifiers • Tested deep learning models (VGG-16, MobileNet) for defect classification • Implemented spatial pyramid matching to capture spatial relationships	• 398 defective and 400 non-defective PV module images from three plants • Defects: block (32%), patchwork (22%), single (22%), string (21%), soiling (3%) • Soiling class excluded due to high variation • Images captured using a FLIR Tau 2 640 camera	• Defect detection: 91.2% accuracy (random forest with SIFT) • Defect classification: 89.5% accuracy (MobileNet with Adam optimiser) • Spatial pyramid matching: 77.1% accuracy • VGG-16: 85.8% accuracy (SGD optimiser)	• Small dataset size, especially for soiling defects • High computational cost for deep learning models • Feature-based methods struggled with similar defect classes (e.g., patchwork vs. string) • Training times for CNNs were long without GPU acceleration
^45^	• Designed a multi-spectral CNN model to detect solar cell surface defects • Analyzed defect features in different spectral bands (R, G, B) • Used sliding window segmentation to create smaller images for training • Compared three CNN structures to select the optimal model	• Collected 15,330 non-defective and 5,915 defective solar cell images • Images split into 469 × 469 pixel blocks using a sliding window • Defects included broken gates, paste spots, dirty cells, thick lines, scratches, and color differences	• Achieved 94.30% defect recognition accuracy • Used precision, recall, and F-measure for evaluation • Conducted fivefold cross-validation for robustness	• Lower detection rates for small or linear defects (e.g., scratches) • Training time for the multi-spectral CNN was longer • Required manual screening of dataset images • Limited to specific defect types in polycrystalline silicon cells
^11^	• The study uses two machine learning approaches for PV defect classification: SVM with feature extraction (HOG, KAZE, SIFT, SURF) and CNN • Hyperparameters, optimizers, and loss functions are tuned for performance	• Electroluminescence (EL) images of PV cells are used, covering seven defect classes (one non-defective, six defective)	• CNN achieves 91.58% accuracy, outperforming SVM with HOG (69.95%), KAZE (71.04%), SIFT (68.90%), and SURF (72.74%)	• SVM performance varies with feature extraction methods • CNN may require more computational resources • Inhomogeneous crack intensity and complex backgrounds challenge classification
^47^	• The paper proposes a pipeline for automatic solar cell classification using electroluminescence (EL) imaging • It focuses on hardware setup, image acquisition, and algorithm evaluation • A representative image batch is created, and reference decisions are made by multiple employees	• A representative batch of ~ 30,000 EL images is assembled by sampling every 50th image over one week from three production lines • The batch includes both acceptable (OK) and defective (nOK) cells with varied defects and severities	• Performance is evaluated using confusion matrices • Key metrics are overkill (false positives) and underkill (false negatives) rates. The algorithm (ELEval-2) outperforms human operators, with lower average overkill (1.29% vs. 2.56%) and underkill (17.34% vs. 18.34%) rates	• The method relies on human reference decisions, which can be inconsistent • The dataset may not capture all defect types if production issues persist • The approach requires significant effort to collect and label images • Performance depends on image quality and hardware constraints
^48^	• Proposed a light CNN architecture for defect detection in PV cell EL images • Conducted extensive experiments with various architectures to optimize performance • Used data augmentation (rotation, flipping, cropping, contrast, blurring) to address data scarcity	• Public solar cell dataset with 2,624 EL images from 44 PV modules • Included mono-crystalline and poly-crystalline cells with defects like cracks, finger failures, and material defects • Images were preprocessed and augmented to enhance model robustness	• Achieved 93.02% accuracy with a standard deviation of 0.37% using four-fold cross-validation • Precision: 0.92 (normal), 0.93 (defective) • Recall: 0.93 (normal), 0.91 (defective) • F1 score: 0.9249 (normal), 0.9198 (defective) • Prediction time: 8.07 ms per image on a non-GPU computer	• Performance drops for poly-crystalline cells due to complex textures • Requires manual labeling for new defect types • Limited by small dataset size, affecting generalization • Misclassifies minor defects and defects like background textures
^49^	PV Cell Segmentation: • Preprocessing—Contrast enhancement + ridge detection • Curve Extraction—Subpixel parabolic fitting of ridges • Grid Modeling—Lens distortion correction via plumbline optimization • Cell Extraction—Topology inference + grid-based cell isolation	• The dataset includes 44 PV modules (26 monocrystalline, 18 polycrystalline) with 2,624 cells, of which 715 are defective • Test images (8 total) contain 408 hand-labeled cells. Image resolutions average ~ 2500 × 2000 pixels	• Root Mean Square Error (RMSE): Measures corner alignment accuracy • Pixelwise Scores: Precision, recall, and F1 score for multiclass segmentation • Weighted Jaccard Index: Evaluates mask similarity, achieving 97.62% F1 score and 94.47% median Jaccard index	• Spurious edges from mounts may cause errors • Disconnected cells (> 50% dark) reduce grid detection accuracy • Smooth or wide inter-cell borders may fail detection • Runtime (~ 66 s per image) may need optimization for field use
^50^	• Used electroluminescence (EL) imaging to analyze PV modules • Developed image processing and machine learning techniques • Extracted features like median intensity and fraction of dark pixels	• 30 commercial 60-cell PV modules from 5 brands • Exposed to damp heat (DH) and thermal cycling (TC) • 195 module-level EL images and 11,700 cell-level images • Included current–voltage (I-V) curves and EL images	• CNN achieved 95% accuracy in classifying busbar corrosion • Power prediction models had adjusted-R^2^ up to 0.88 • Series resistance prediction models had adjusted-R^2^ up to 0.73	• Required initial module characterization for normalization • Models were tested only on specific brands and exposures • Limited to lab conditions; field validation needed • Some features were only applicable to certain degradation types
^51^	• The paper proposes AC-PG GAN, a GAN-based model combining Progressive Growing GAN (PGGAN) and Auxiliary Classifier GAN (ACGAN) to generate synthetic EL images • The model uses progressive training and label guidance to improve sample quality • Three CNN models (AlexNet, ResNet, SqueezeNet) are tested for classification	• The dataset includes 507 EL images of monocrystalline solar cells with four defect types: grid fingers (312), material defects (30), microcracks (99), and deep cracks (66) • Traditional augmentation (flips, rotations, color jittering) expands the dataset 11x	• Classification accuracy is measured for each defect type. ResNet performs best (up to 100% accuracy) • AlexNet shows the highest improvement (14%) with GAN-augmented data • SqueezeNet performs the worst	• Training GANs is unstable and computationally expensive • Generated deep crack images reduce microcrack classification accuracy due to visual similarities • The method requires careful tuning and may not scale easily for large datasets
^52^	• The paper proposes a solar cell micro-crack detection system using electroluminescence (EL) imaging • It combines a healthy solar cell image with a cracked one using an OR function to enhance crack visibility	• The study uses real solar cell images captured with an EL system • Images include healthy and cracked cells, with resolutions of 200 × 200 and 300 × 300 pixels • Some images were taken under electron microscopy at magnifications of 10 μm to 1 mm	• Crack detection accuracy is based on gray level thresholds (254 ± 10%) • The method identifies crack size (200–700 μm), location, and orientation • It compares results with conventional EL imaging	• The method cannot measure crack depth • It requires manual calibration for plot profiles • Detection of point cracks (< 250 μm) is less accurate • Performance depends on image resolution and biasing current
^19^	• Two approaches: SVM with hand-crafted features and CNN for defect classification in PV cells • SVM uses KAZE/VGG features, VLAD encoding, and linear/RBF kernels • CNN fine-tunes Vgg-19 with transfer learning, global average pooling, and regression output • Data augmentation (scaling, rotation, flipping) applied for CNN training	• 2,624 EL images of mono- and polycrystalline PV cells (300 × 300 pixels) • Labels: functional (0%), defective (100%), and uncertain (33%, 67%) based on expert assessment • Split: 75% training (1,968 cells), 25% testing (656 cells)	• SVM: 82.44% accuracy, 82.52% F1 score • CNN: 88.42% accuracy, 88.39% F1 score • ROC AUC: CNN outperforms SVM, especially for polycrystalline cells	• Independent cell analysis ignores contextual defects across modules • Small dataset limits generalization: performance improves logarithmically with more data • SVM is slower than CNN on CPU; CNN requires a GPU for efficient inference • Finger interruptions are confused with defects; need more labeled data for better distinction
^53^	• Proposed a deep-learning-based method for PV cell defect detection using EL images • Used data augmentation and category weight assignment to handle small, imbalanced datasets • Fused ResNet152 and Xception models for enhanced feature extraction • Incorporated Coordinate Attention (CA) mechanism to improve accuracy	• Dataset 1: 2,624 EL images (300 × 300 pixels), labeled as"0%"(no defect) or"100%"(defect) • Dataset 2: High-resolution images with 10 defect types, including cracks, broken grids, and black cores • Split datasets into 80% training and 20% testing	• Achieved 96.17% accuracy in binary classification (defect vs. no defect) • Achieved 92.13% accuracy in multiclass classification (10 defect types) • Outperformed CNN, VGG16, MobileNetV2, and other models in accuracy, F1 score, recall, and precision	• Relies on limited public datasets; may not generalize to all PV cell defects • Requires high computational resources for training • Performance may drop for extremely subtle defects • Data imbalance remains a challenge despite weighting strategies
^54^	• The paper proposes an end-to-end deep learning pipeline for detecting, locating, and segmenting cell-level anomalies in PV modules using EL images • The pipeline consists of four modules: • Detection: Uses a modified Faster R-CNN with ResNet101 to extract PV cells from module images • Classification: Employs EfficientNet-B1 to classify cells as defective or non-defective	• ELPV Dataset: Contains 2,624 grayscale images (300 × 300 pixels) of monocrystalline and polycrystalline cells, labeled by defect likelihood • TecnaliaPR Dataset: Includes 67 EL images of entire PV modules (2111 × 1261 pixels) with 5,592 annotated cells of varying types (3/5 busbars, elongated) • Both datasets were augmented to balance classes and improve model robustness	• Detection: Achieved 99.36% average precision (AP) in locating cells • Classification: Attained 84% accuracy in distinguishing defective from non-defective cells • Segmentation: Autoencoder achieved 0.992 structural similarity index (SSIM) for anomaly segmentation	• Data Diversity: Limited to specific PV cell types; performance may degrade with unseen cell technologies • Segmentation Precision: Struggles with thin cracks or cells containing multiple anomalies • Preprocessing Dependency: Requires prior image rectification (e.g., distortion correction) not included in the pipeline • Computational Cost: Training deep learning models demands significant resources
^55^	• Combines weakly-supervised deep autoencoder and unsupervised clustering (DBSCAN, alpha-shape) • Uses SSIM for disparity maps. Adaptable to new PV cell types	• Public ELPV dataset (mono-crystalline cells). 588 non-defective images, augmented to 2352. Private dataset with 5-busbar cells	• Reduced annotation cost by 60% (7.5 s/image vs. 19.9 s manually) • SSIM accuracy: mean 0.94, median 0.95	• Noisy disparity maps near busbars/corners. Poor performance on poly-crystalline cells • Requires tuning for new cell types. Manual review needed for Gold Standard
^56^	• The paper proposes an automatic cell segmentation and defect detection system (SCDD) for electroluminescence (EL) images of solar panels • It uses contour tracing and probabilistic Hough transform for cell segmentation, followed by CNN-based defect detection (YOLOv4) and pseudo-colorization (K-means clustering) for defect visualization	• The dataset includes 96 training and 23 testing EL panel images, each containing 60 cells (total 7,140 cells). Defect annotations were manually labeled for training. Data augmentation (flipping) was applied to increase variability	• Cell segmentation errors: 1.6 pixels (x-direction), 1.4 pixels (y-direction) • Defect detection accuracy: 99.8% (YOLOv4) • Pseudo-colorization improved defect visibility	• Segmentation speed (~ 2.71 s per image) needs optimization • Tiny defects near cell boundaries may be missed • Limited to single-crystalline silicon PV modules • Requires manual annotation for training data
^57^	• Used DCGAN to generate synthetic EL images of PV cells • Preprocessed real EL images to remove noise and normalize lighting • Trained a Random Forest model to predict energy output for synthetic images • Validated results using visual analysis and quality metrics	• Original dataset: 602 real EL images with IV curves • Synthetic dataset: 10,000 generated EL images • Images labeled by relative power output (Class 0: ≥ 0.8, Class 1: < 0.8) • Publicly available on GitHub	• Inception Score (IS): 2.3 for synthetic images (close to real images’ 2.1) • Fréchet Inception Distance (FID): 15.8 between real and synthetic images • Random Forest MAE: 0.041, MSE: 0.0038 for power prediction	• Small original dataset (602 images) • Synthetic images lack extreme intensity values found in real images • Limited variety of defects in original data affects synthetic diversity • Hardware constraints limited model size and training epochs
^58^	• Proposed a two-step deep learning method for solar cell binning using electroluminescence (EL) images • Step 1: Trained CNNs (AlexNet, ResNet, SqueezeNet, VGGNet) • Step 2: Used CNN-extracted features with ML regressors (RF, AdaBoost, SVM) to predict cell efficiency • Introduced a Universal CNN for transfer learning and fine-tuning	• Three datasets: M0 (busbar-less), M3 (3-busbar), M5 (5-busbar) with ~ 20,000 EL-I-V pairs each • Standardized efficiency to median 20% and mean absolute deviation 0.3% • Binned into“reject”(< 19%) and ten 0.2%-wide bins (19%−21%)	• Classification: Cross-entropy loss (CEL),“reject”accuracy (~ 80%),"non-reject"accuracy (~ 60%) • Regression: R^2^ (up to 0.93), RMSE (< 0.1% absolute efficiency) • Mismatch loss difference between I-V and CNN + ML binning: < 0.002%	• Low“reject”accuracy due to small dataset size (~ 2% reject cells) • Sharp binning thresholds caused misclassification despite low RMSE • RGB superposition for half-cells required larger datasets for optimal performance • Assumed short-circuit current as proxy for maximum power point current
^59^	• The paper proposes a hybrid system using Inception-V3 and ResNet50 to extract deep features from EL images • Features are fused and classified using sigmoid (binary) or Softmax (multi-class) activation. Data augmentation (rotation, flipping, blurring) is applied to prevent overfitting	• A public dataset of 2,624 EL images (300 × 300 pixels) was used. Images were labeled as functional, mild, moderate, or severe defects • The dataset was split into 80% training and 20% testing	• The system achieved 98.15% accuracy (binary) and 95.35% (multi-class) • Other metrics included recall, precision, F1-score, and specificity • ROC curves confirmed model robustness	• EL images contain noise, reducing clarity • The model needs more diverse data for generalization • Similar textures in normal and defective cells cause classification challenges • Training requires significant computational resources

## Materials and methods

### Dataset

The researchers introduced a publicly available dataset of solar cell images^26^, comprising high-resolution electroluminescence (EL) captures from both monocrystalline and polycrystalline photovoltaic modules^60^. Each solar cell image has a resolution of 2624 pixels (300 × 300). The dataset comprises images collected from 44 distinct PV modules (18 monocrystalline and 26 polycrystalline types). The PV module images were captured under controlled conditions at the production facility to minimize detrimental effects like overexposure. These controlled settings were necessary as background irradiation could dominate EL irradiation. Imaging in a dark room ensured even lighting, with only the PV module generating light. For classification purposes, the extracted cells were randomly presented to experts familiar with various flaws in EL images. These experts focused on known faults causing more than 3% power loss from initial output, following principles summarized in^16^. The experts evaluated: (1) whether the cell was functional or faulty, and (2) their confidence in this assessment. The confident assessors’ evaluations were used directly as labels. For less confident assessors, all cells (both functional and defective) were labeled as defective but with different weights: 33% weight for uncertain functional cell ratings and 67% weight for uncertain defective cell ratings. Table 2 shows the evaluators’ ratings and corresponding labels and weights.

**Table 2:**
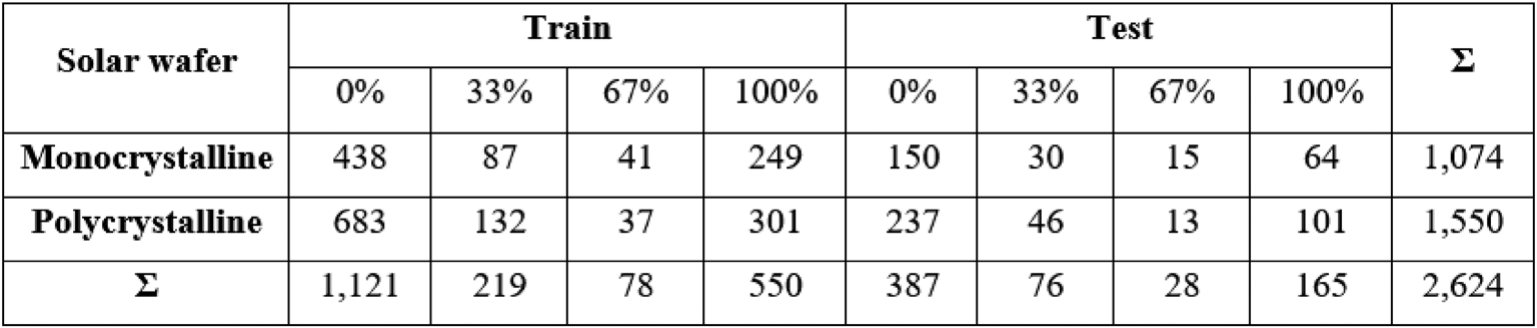
Functional and defective solar cell classification with self-assessment.

Table 3 displays the distribution of labeled solar cells by PV module type. The dataset was split with 25% (656 cells) for testing and 75% (1968 cells) for training. Stratified sampling maintained the class distribution in both sets. The dataset consists of eight classes: four representing defect percentages (0%, 33%, 66%, and 100%) and four representing solar cell types (monocrystalline or polycrystalline) for each defect percentage.

**Table 3:**
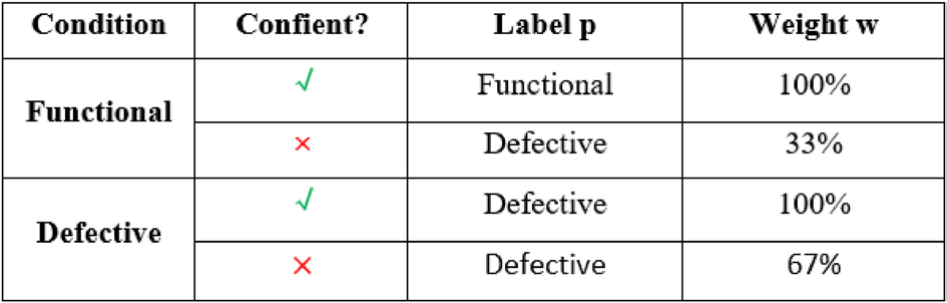
Summary of solar cell image dataset: distribution by module type, sample label, and train-test split.

### Preprocessing and data augmentation

**Fig. 2:**
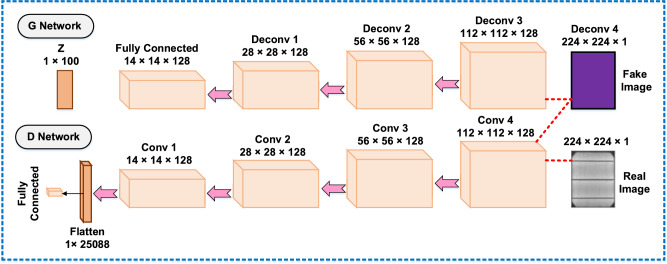
GAN model structure.

Large datasets are crucial for optimizing deep learning model performance and preventing overfitting. While increasing dataset size generally enhances model accuracy, practical constraints often necessitate data augmentation techniques such as cropping, rotation, noise injection, and image inversion^50,60^. In this study, limited training data prompted dataset expansion through offline augmentation methods. These techniques proved particularly suitable for EL image analysis since defect orientation does not impact classification outcomes in solar cell fault detection. The research team developed a novel GAN-based augmentation framework comprising:

1. A generative network (G-network) with:
  ○ Four transposed convolutional layers
  ○ One fully connected layer that:
    ■ Processes 100-dimensional noise vectors as input
    ■ Transforms them into (16*16*512) feature tensors
    ■ Utilizes 256, 128, 64, and 1 convolutional kernels for local feature extraction
    ■ Generates synthetic (256*256*1) EL images
2. A discriminative network (D-network) for adversarial training (as illustrated in Fig. 2)

The D-network consists of 4 convolutional layers and a fully connected layer that determines whether an image is real or fake. Table 3 lists the parameters for both network structures. The proposed model uses a G-network that takes noise as input and produces fake images as output. The D-network then distinguishes between generated and real images. The Earth Mover (EM) distance measures the difference between generated and real images, with cost functions for the G-network and D-network defined by Eqs. (1) and (2).

1 $${G}_{loss}=-{E}_{x}- {p}_{g}[ {f}_{w}\left(x\right) ]$$

2 $${D}_{loss}=-{E}_{x}- {p}_{g}\left[ {f}_{w}\left(x\right) \right]-{E}_{x}-{p}_{r}\left[ {f}_{w}\left(x\right)\right]$$

${\text{f}}_{\text{w}}\left(\text{x}\right)$ where symbolizes the D-network model, with input x coming from either generated or actual pictures. RMS prop (Root Mean Square Prop) was selected as the optimization technique for training the model; ${D}_{loss}$ should be minimized to approximate the Wasserstein distance; the initial hyperparameters of GAN are exposed in Table 5. Matched with traditional image processing methods, GAN-based image processing requires more computational time and has moderately high demand on computer hardware. EL images of dissimilar defects generated using the GAN model are shown in Fig. 2. This method has advantages over the data enhancement method: GAN can generate new images, GAN can extract deep picture characteristics for image improvement, and GAN can be used to enhance the image of defects.

The enhanced images generated through this process exhibit substantial qualitative improvements that can potentially enhance CNN model performance during training. However, several critical considerations must be addressed: (1) training dataset size must be carefully optimized to prevent overfitting, and (2) the inherent stochasticity of GAN-based image generation requires particular attention. To address data scarcity challenges, we implement a two-stage augmentation pipeline where the GAN model first generates synthetic samples, which are then combined with the original dataset for subsequent CNN training. This hybrid approach significantly improves augmentation efficiency in data-limited scenarios. In our study, we implemented a GAN-based oversampling strategy referred to as AUG300, in which 300 synthetic images were generated per class, regardless of the original class balance. Unlike traditional methods such as SMOTE, which operate in feature space using interpolation between existing samples, our approach leverages the ability of GANs to generate entirely new, high-resolution electroluminescence images with realistic defect patterns. This strategy not only enhances the diversity of the dataset but also introduces a controlled augmentation volume that helps counteract class imbalance and model bias. By enriching all classes equally and consistently, AUG300 contributed to improving classification robustness and generalization in our deep learning models.(Table 4).

**Table 4:** GAN model architecture parameters.

G-network	Output shape	Parameters	D-network	Output shape	Parameters
Full connected	14 × 14 × 128	2,544,284	Convolution layer 1#	224 × 224 × 1	2,559,376
Deconvolution layer 1#	28 × 28 × 128	264,320	Convolution layer 2#	112 × 112 × 128	2432
Deconvolution layer 2#	56 × 56 × 128	262,272	Convolution layer 3#	56 × 56 × 128	147,584
Deconvolution layer 3#	112 × 112 × 128	262,272	Convolution layer 4#	28 × 28 × 128	147,584
Deconvolution layer 4#	224 × 224 × 128	262,272	Convolution layer 5#	14 × 14 × 128	147,584
Deconvolution layer 5#	224 × 224 × 1	6273	Flatten	25,088	0
			Full connected	1	25,089

**Fig. 3:**
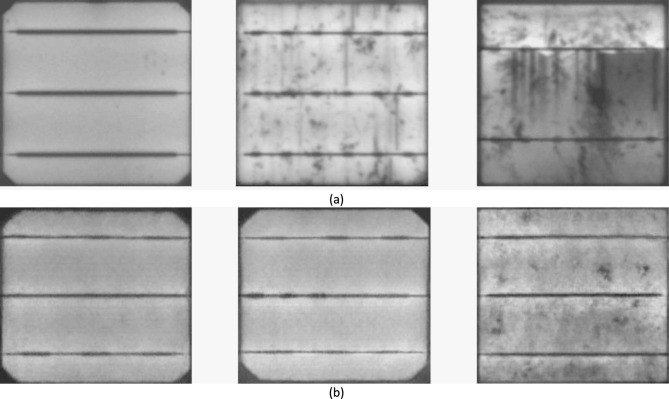
Visual comparison between (**a**) real and (**b**) GAN-generated electroluminescence (EL) images.

To ensure the physical realism and class-specific consistency of the GAN-generated EL images, multiple validation steps were performed. A visual inspection was conducted by comparing synthetic images with real EL samples, highlighting their similarity in crack structures, brightness levels, and texture patterns. As illustrated in Fig. 3, the generated images demonstrate strong visual alignment with real data across different defect types. In addition, a pretrained classifier was used to evaluate the semantic consistency of the generated images, and only high-confidence samples were included in the final dataset. Statistical analysis of pixel intensity distributions further confirmed the alignment between real and synthetic data, supporting the reliability of the GAN-augmented dataset for model training.

### One-cycle policy

The one-cycle strategy proves highly effective when training complex models, delivering rapid results. It leverages the Cyclical Learning Rate (CLR) to achieve faster training times while providing regularization benefits with minimal modifications. By selecting optimal learning rates at each iteration, the model can converge quickly.

This strategy implements a cycle shorter than the total number of iterations/epochs, allowing the learning rate to decrease by several orders of magnitude below the initial rate during remaining iterations. The CLR philosophy effectively combines curricular learning with simulated annealing approaches. For certain hyper-parameter values, using extremely high learning rates with the CLR approach can dramatically accelerate training—up to ten times faster. This remarkable acceleration phenomenon was termed"Super-Convergence"by Leslie Smith^61^.

### Changing learning rates

According to the research^62^, a cycle with two equal-length steps is recommended, with the first step increasing from a lower to a higher learning rate and the second step returning to a minimum. It is worth noting that the growth and reduction change in a linear fashion. The maximum learning rate should be determined using the learning rate finder tool. For the minimum learning rate, it can be set to approximately 1/3 or 1/4 of the maximum rate. The lowest learning rate should then be reduced further by a factor of 10 from this minimum value. The cycle length should be configured to be shorter than the total number of training epochs. This structure ensures that the final epochs will operate with a learning rate that’s several orders of magnitude lower than even the lowest specified rate in the cycle, allowing for fine-tuning and convergence at the end of training.

### Architecture for deep transfer learning

Deep learning is a ML subfield inspired by brain structure. In recent years, these approaches have shown exceptional performance in PV cell image processing. Applying deep learning algorithms to PV data aims to extract valuable insights. These models have successfully been used across various applications including classification, segmentation, and lesion identification in PV cell data.

PV cell imaging techniques like IR imaging and EL imaging are analyzed using deep learning models to examine image and signal data. These investigations help detect and categorize defects including micro-cracks, finger failure, silicon material defects, cell connectivity deterioration, and electrical separation or recognition problems^63^.

In convolutional neural network (CNN) processing, the system first encodes input images into numerical matrices for computational processing. This matrix representation enables the network to establish correlations between image transformations and their corresponding labels. Through iterative training, CNN learns to associate specific spatial patterns with classification outcomes, building predictive capabilities for new images.

The fundamental CNN architecture comprises three core components arranged in sequence^64^:

1. **Convolutional Layers**: Perform feature extraction through learned filters that detect spatial hierarchies of patterns
2. **Pooling Layers**: Reduce dimensionality while preserving critical features through operations like max-pooling
3. **Fully Connected Layers**: Integrate extracted features for final classification decisions

This layered architecture progressively transforms raw pixel data into increasingly abstract representations, enabling effective pattern recognition while maintaining spatial relationships within the input data.

#### Convolutional layer

The convolutional layer constitutes the fundamental building block of CNN architectures, performing localized feature extraction through learned filter operations. These layers systematically identify and quantify distinctive spatial patterns by computing dot products between small receptive fields and convolutional kernels (filters). The process generates feature maps that encode hierarchical representations of the input data, with early layers capturing basic visual elements (edges, textures) and deeper layers detecting increasingly complex patterns. When an image is provided as input, this layer applies filters to process it. The filtering operation produces values that collectively form a feature map. Within this layer, kernels (small matrices) slide across the pattern to capture both simple and complex information^65^. These kernels, typically sized as 3 × 3 or 5 × 5 matrices, transform the input pattern through matrix operations. The stride parameter defines how many steps the kernel takes when moving across the input matrix. The result of the convolutional layer may be stated as:

3 $${X}_{j}^{l}=f(\sum_{a=1}^{N}{w}_{j}^{l-1}*{y}_{a}^{l-1}+{b}_{j}^{l})$$

where ${X}_{j}^{l}$ is the ${j}^{th}$ feature map in layer l, ${w}_{j}^{l-1}$ indicates jth kernels in layer $l-1$, ${y}_{a}^{l-1}$ represents the ath feature map in layer $l-1$, ${b}_{j}^{l}$ indicates the bias of the $jth$ feature map in layer l, N is the number of total features in layer $l-1$, and (∗) represents the vector convolution process^64^.

#### Pooling layer

Following the convolutional layer is the pooling layer. This layer’s purpose is to decrease the number of feature maps and network parameters by applying specific mathematical operations. The research^66^ employs both maximum pooling and global average pooling techniques. The max-pooling operation downsamples feature maps by extracting only the maximum activation value within each n*n sliding window (typically 2*2), producing more compact representations while preserving the most salient features. This spatial reduction (1) decreases computational complexity and (2) provides basic translation invariance. The architecture incorporates two additional critical layers:

1. **Global average pooling (GAP)**:
  o Replaces flattening operations before the fully connected layer
  o Reduces each feature map to its spatial meaning
  o Generates 1D feature vectors while maintaining spatial relationships
2. **Dropout layer**:
  o Randomly deactivates neurons during training (typically with p = 0.5)
  o Creating an implicit ensemble effect
  o Effectively regularizes the network by preventing co-adaptation of features

#### Fully connected layer

The fully connected layer represents the final and most crucial component of CNN architecture. This layer operates as a multilayer perceptron. Within fully connected layers, the rectified linear unit (Relu) activation function is commonly implemented, while the SoftMax activation function is applied to predicted output images in the final layer of the fully connected section. The mathematical formulations for these two activation functions would follow:

4 $$\text{Relu}\left(\text{x}\right)=\left\{\begin{array}{c}0,\quad if \ x<0\\ x, \quad if x \ \ge 0\end{array}\right.$$

5 $$\text{Softmax}\left(\text{x}\right)=\frac{{\text{e}}^{{\text{x}}_{\text{i}}}}{\sum_{\text{y}=1}^{\text{m}}{\text{e}}_{\text{y}}^{\text{x}}}$$

where ${x}_{i}$ and $m$ represent the input data and the number of classes, respectively. Within a fully connected layer, each neuron maintains complete connections to all activation functions from the preceding layer.

#### Pre‑trained models

Training convolutional neural networks (CNNs) with millions of parameters from scratch demands significant computational resources and time. To mitigate these challenges, transfer learning has emerged as an effective strategy, where knowledge (weights and parameters) from models pre-trained on large datasets (e.g., ImageNet) is transferred to new tasks^67,68^. This approach focuses learning on newly added task-specific layers while preserving the transferred feature extraction capabilities, significantly reducing training time and computational costs—particularly beneficial for limited datasets^69^. A critical challenge in photovoltaic (PV) cell analysis is dataset scarcity. While deep learning traditionally requires extensive labeled data, manual annotation is costly and labor-intensive. Transfer learning addresses this by leveraging pre-trained models’ learned features, enabling effective training even with smaller datasets.

**Fig. 4:**
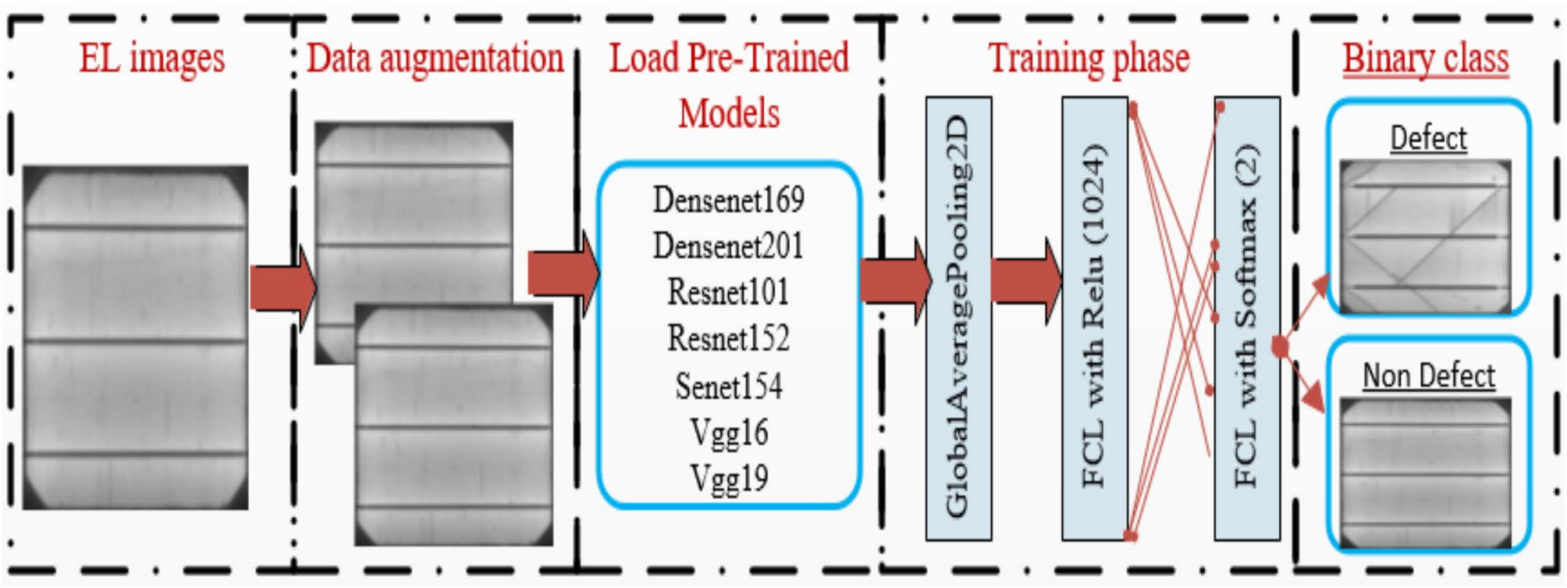
Architecture of pre-trained models for defect and Non-defect EL image prediction, illustrating the overall system workflow.

In this study, we implemented deep CNN-based architectures—including DenseNet169, DenseNet201, ResNet101, ResNet152, SENet154, VGG16, and VGG19—to classify electroluminescence (EL) images of PV cells into defective and non-defective categories. To further compensate for data limitations, we integrated generative adversarial network (GAN)-based augmentation, synthetically expanding the training dataset. Figure 4 illustrates the proposed framework, which combines pre-trained models with augmented data for optimized defect detection. In addition to showing model architecture, Fig. 3 also summarizes the overall system workflow, including input, augmentation, model training, and classification output, providing a clear visual overview of the proposed methodology.

##### Densenet-169 and densenet201

DenseNet-169 and DenseNet-201 are part of the DenseNet model family, widely used for image classification tasks. The primary distinction between DenseNet-201 and other models in the series lies in their size and accuracy. DenseNet-201 is marginally larger, with a size of approximately 77MB, compared to DenseNet-169, which is around 55MB. Initially trained using Torch, these models were later converted to the Caffe* framework. All DenseNet variants were pre-trained on the ImageNet image dataset. The models accept input as a blob representing a single image with dimensions 1 × 3 × 224 × 224 in BGR format. Before feeding the image into the network, the mean BGR values [103.94, 116.78, 123.68] should be subtracted, and the resulting values should be scaled by dividing by 0.017. Both DenseNet-169 and DenseNet-201 generate standard classification outputs across 1000 categories, consistent with the ImageNet dataset classifications^70,71^.

##### Senet154

SENet-154 is an upgraded version of 64*4d ResNeXt-152 that incorporates SE blocks and distributes the original ResNeXt-101 using ResNet-152’s block stacking approach^72,73^.

##### Vgg16 and Vgg19

Vgg16 and Vgg19 are both convolutional neural network architectures with minimal differences between them apart from their layer depth. The VGG-16 model represents a convolutional neural network that underwent training on more than one million images from the ImageNet database. This network contains 16 layers and can classify images across 1000 different object categories, including keyboards, mice, pencils, and various animals. The network requires input images with dimensions of 224 by 224 pixels. Vgg19 follows the same architectural principles as Vgg16, with the primary distinction being its increased depth of 19 layers. Regardless of the specific version, both networks analyze image objects using convolutional neural network methodology^68,74^.

##### ResNet101 and ResNet152

The architectures of ResNet101 and ResNet152 contain 101 and 152 layers respectively, achieved through layered ResNet building blocks. A pre-trained version of this network exists in the ImageNet database, having undergone training on more than a million images. This extensive training has enabled the network to develop sophisticated feature representations applicable to diverse image types. The network requires input images sized at 224 × 224 pixels^68,75^.

### Experimental setup

The implementation of the proposed deep transfer learning models was conducted using Python programming language. All experimental testing was performed on a Google Collaboratory (Colab) Linux server running Ubuntu 16.04 operating system, utilizing the free online cloud service with hardware options including Central Processing Unit (CPU), Tesla K80 Graphics Processing Unit (GPU), or Tensor Processing Unit (TPU). For the GAN hyperparameters shown in Table 5, an optimal batch size of 32 was selected for the GAN model, along with 100 epochs and a 0.0002 learning rate. The CNN architectures (Densenet169, Densenet201, Resnet101, Resnet152, Senet154, Vgg16, and Vgg19) underwent pre-training with random initialization weights by optimizing the cross-entropy function using the adaptive moment estimation (ADAM) optimizer (with parameters β₁ = 0.9 and β₂ = 0.999). ReLU activation functions were implemented throughout all convolutional layers.

**Table 5:** Summary of initial hyperparameters for GAN training.

Hyper-parameters	Value
Batch size	32
Epoch	100
Learning rate	0.0002
Beta_1	0.5

In one approach, we used a batch size of 16 with a learning rate of 0.004, while in the alternative approach, the learning rate was determined by the“find_lr”function from the fastai Python library. For all experiments, the number of epochs was empirically set at 70. The datasets were randomly split into two segments: 80% allocated for training and 20% reserved for testing.

### Performance evaluation metrics

To assess the algorithms’ performance, the following measures are used:

• **Recall** is a function of both successfully categorized instances (Tp) and erroneously classed examples (Fn).
  6 $$recall= \frac{{t}_{p}}{{t}_{p}+{f}_{n}}$$

where ${t}_{p}$ is the number of true positives and ${f}_{n}$ the number of false negatives.

**• Precision** is a function of actual positives vs cases that were wrongly labeled as positives.
  7 $$Precision= \frac{{t}_{p}}{{t}_{p}+{f}_{p}}$$
**• F1-score** is an amount of a test’s accuracy.
  8 $$F1-score= \frac{\left({\beta }^{2}+1\right)*precision *recall}{{\beta }^{2}*precision *recall}$$

At β = 1, the F1-score is evenly balanced. It prioritizes accuracy when β > 1 and recall otherwise. The F1-score may be seen as a weighted average of accuracy and recall^45,64^.

## Experimental results

We have four classes that can be used in regression or classification problems, we tried also splitting each class from the four classes into our main two types of cells (Monocrystalline and polycrystalline) to get eight classes shown in Table 8, we tested the classification problem over our data which lead to results that could not be used so we apply the regression problem over the data then we apply a threshold that shows if our cell is a defect (class2) or not defect (class1), the threshold, we could get it through analysis over the validation data which show that our model biased to class one over class two so we choose a better-fixed threshold form the analysis over the validation data and apply it to all models in the test phase and this threshold is the result of the mean of the best threshold of our approach models as each model has its own best threshold.

we perform two experiments, the first one with the ability to change the learning rate of the model dynamically using the fastai-lib method “find_lr” which tests multiple learning rates and shows how the loss changes through the different learning rates to choose the best learning rate for the model, the other one we just set a fixed learning rate "1e-4", results of this method called “before experiment” and the first one called" after experiment", and experiment indicates change learning rate dynamically use “find_lr” method to choose best lr for each unique model.

### Regression to classification

We used Regression models instead of classification models as when we tested the classification models, we got unsatisfactory results. We used the main idea of research^50^ as we used the regression models with dynamic thresholds based on analysis of validation data results to create two classes which are defect or non-defect where defect indicates defect more than or nearly to 66% defect and non-defect class indicates less than or nearly to 33% defect.

In Tables 6 and 7, we can see all our model results but using evaluation methods of regression domain like MSE (mean squared error), RMSE (root mean squared error), and MAE (mean absolute error). Before we used a threshold based on the analysis of the models on the validation data, the results were too biased to class one with a big difference, and still, some models in our results after we applied the new threshold have biased results which can be seen in VGG models so after seeing the best models that new threshold creates it shows that in such problems with regression using threshold of 0.5 blindly may lower your true results significantly. To further ensure that threshold tuning did not introduce overfitting, all threshold values were optimized based solely on validation set performance, and no adjustments were made using training data directly. This strategy helped maintain generalization across unseen test data.

**Table 6:** Regression training models before the experiment.

Approaches		CNN models						
		Densenet169	Densenet201	Resnet 101	Resnet 152	Sesnet 154	Vgg16	Vgg19
4 C—AUG	MSE	0.050019	0.045656	0.046934	0.047437	0.077075	0.069834	0.070205
	RMSE	0.216362	0.204020	0.208739	0.210701	0.270117	0.256865	0.258081
	MAE	0.144099	0.132143	0.135859	0.139979	0.187212	0.186722	0.178798
4C—AUG300	MSE	0.049280	0.050540	0.045175	0.052016	0.070284	0.070577	0.063277
	RMSE	0.214799	0.220147	0.206018	0.218814	0.256892	0.258703	0.244627
	MAE	0.140635	0.138833	0.134138	0.138517	0.182426	0.178444	0.177594
8C—AUG	MSE	0.048634	0.059338	0.056579	0.053408	0.073402	0.069793	0.071593
	RMSE	0.210108	0.232592	0.231885	0.224434	0.265398	0.258614	0.261194
	MAE	0.137138	0.147851	0.160756	0.147844	0.195760	0.196075	0.192256
8C—AUG300	MSE	0.049723	0.047648	0.049054	0.051563	0.071324	0.070262	0.080236
	RMSE	0.212561	0.207399	0.217119	0.219002	0.260122	0.259357	0.275956
	MAE	0.138427	0.139214	0.146617	0.140718	0.185616	0.191407	0.191182

**Table 7:** Regression training models after experiment.

Approaches		CNN models						
		Densenet169	Densenet201	Resnet 101	Resnet 152	Sesnet 154	Vgg16	Vgg19
4 C—AUG	MSE	0.045874	0.104299	0.068607	0.058174	0.077075	0.131920	0.065414
	RMSE	0.203550	0.317011	0.256295	0.233995	0.270117	0.327588	0.247575
	MAE	0.132075	0.234504	0.180098	0.159561	0.187212	0.202792	0.164451
4C—AUG300	MSE	0.054814	0.088152	0.044780	0.052026	0.070284	0.070565	0.056270
	RMSE	0.224388	0.289548	0.201740	0.214046	0.256892	0.252800	0.228797
	MAE	0.138746	0.212875	0.137503	0.133225	0.182426	0.176988	0.157953
8C—AUG	MSE	0.055051	0.087869	0.058993	0.050161	0.073402	0.063205	0.085628
	RMSE	0.225554	0.291325	0.234906	0.217868	0.265398	0.244644	0.271598
	MAE	0.152113	0.225932	0.160301	0.154511	0.195760	0.175017	0.188634
8C—AUG300	MSE	0.043270	0.050245	0.055276	0.070687	0.071324	0.070262	0.077834
	RMSE	0.200542	0.211408	0.228822	0.259007	0.260122	0.259357	0.266626
	MAE	0.137304	0.139210	0.158047	0.202167	0.185616	0.191407	0.193008

### Results before the experiment

Table 8 presents the results of four different approaches for predicting defects and non-defects in solar cells. Each approach is characterized by the number of classes used in training and whether balanced augmentation was applied. Additionally, some approaches increased the number of weakly predicted classes by 300 to address the imbalance problem in our dataset. The results indicate that the 8C_AUG300 approach, which uses eight classes with GAN augmentation and increases weak predicted classes by 300, achieves the best overall performance. This approach yields the highest maximum recall value and is also near the best average recall across all models. Within the 8C_AUG300 approach, the DenseNet201 model stands out with exceptional performance, achieving a recall of 90.58% for the“not defect”class and 88.41% for the“defect”class. This balanced score demonstrates that our best model is not biased towards either class, likely due to the increased number of weakly predicted classes.

**Table 8:** Models evaluation by class recall mean.

CNN models	Approaches			
	4 C—AUG	4C—AUG300	8 C—AUG	8C—AUG300
densenet169	**0.8892**	0.875	0.8645	0.8761
densenet201	0.8867	0.88	0.8612	**0.895**
resnet101	**0.8728**	0.8631	0.8606	0.8717
resnet152	**0.8858**	0.8673	0.8601	0.8592
senet154	0.8107	0.8371	0.834	**0.8426**
vgg16	0.8329	0.8129	0.8077	**0.8443**
vgg19	0.816	**0.819**	0.8182	0.8041
Mean	**0.8563**	0.8506	0.8437	0.8561
Max	0.8892	0.88	0.8645	**0.895**
Min	0.8107	**0.8129**	0.8077	0.8041

**Fig. 5:**
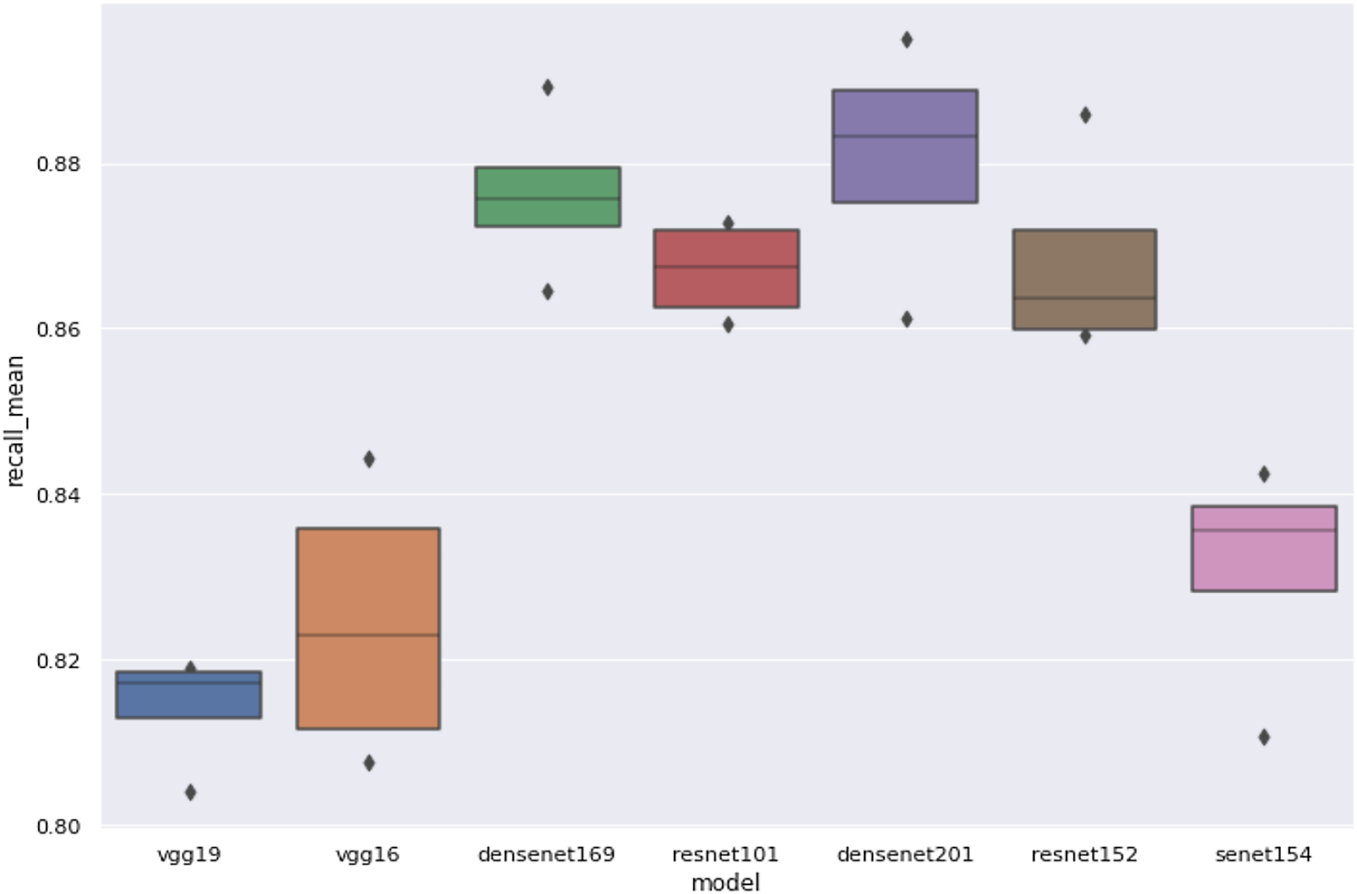
Models evaluation by class recall mean before LR changes.

As shown in Fig. 5, the DenseNet201 model in the 8C_AUG300 approach consistently outperforms other models, even in the“before experiment”stage. While other models also show strong results, DenseNet201 consistently demonstrates superior performance, making it the best single model overall. Tables 9, 10, 11, 12 provide a detailed evaluation of each model’s precision, recall, and F1-score for both classes in each approach. This analysis further confirms the superiority of the 8C_AUG300 approach and the DenseNet201 model.

**Table 9:** F4C AUG evaluation.

CNN models	Matrices					
	Precision		Recall		F1-Score	
	class 1	class 2	class 1	class 2	class 1	class 2
densenet169	0.9307	0.8476	0.9307	0.8476	0.9307	0.8476
densenet201	0.9326	0.8284	0.9197	0.8537	0.9261	0.8408
resnet101	0.9199	0.8282	0.9224	0.8232	0.9212	0.8257
resnet152	0.937	0.8068	0.9058	0.8659	0.9211	0.8353
senet154	0.8655	0.8473	0.9446	0.6768	0.9033	0.7525
vgg16	0.895	0.773	0.8975	0.7683	0.8963	0.7706
vgg19	0.8727	0.8214	0.9307	0.7012	0.9008	0.7566
Mean	0.9076	0.8218	0.9216	0.7909	0.9142	0.8042
Max	0.937	0.8476	0.9446	0.8659	0.9307	0.8476
Min	0.8655	0.773	0.8975	0.6768	0.8963	0.7525

**Table 10:** F4C AUG300 evaluation.

CNN models	Matrices					
	Precision		Recall		F1-Score	
	**class 1**	**class 2**	**class 1**	**class 2**	**class 1**	**class 2**
densenet169	0.9266	0.807	0.9086	0.8415	0.9175	0.8239
densenet201	0.9339	0.7966	0.9003	0.8598	0.9168	0.827
resnet101	0.9183	0.7941	0.903	0.8232	0.9106	0.8084
resnet152	0.9091	0.8609	0.9418	0.7927	0.9252	0.8254
senet154	0.8959	0.7875	0.9058	0.7683	0.9008	0.7778
vgg16	0.8705	0.8201	0.9307	0.6951	0.8996	0.7525
vgg19	0.8832	0.7707	0.9003	0.7378	0.8916	0.7539
Mean	0.9053	0.8053	0.9129	0.7883	0.9089	0.7955
Max	0.9339	0.8609	0.9418	0.8598	0.9252	0.827
Min	0.8705	0.7707	0.9003	0.6951	0.8916	0.7525

**Table 11:** F8C AUG evaluation.

CNN models	Matrices					
	Precision		Recall		F1-Score	
	**class 1**	**class 2**	**class 1**	**class 2**	**class 1**	**class 2**
densenet169	0.9086	0.8497	0.9363	0.7927	0.9222	0.8202
densenet201	0.9139	0.8061	0.9114	0.811	0.9126	0.8085
resnet101	0.9098	0.8239	0.9224	0.7988	0.9161	0.8111
resnet152	0.9157	0.7929	0.903	0.8171	0.9093	0.8048
senet154	0.8934	0.7862	0.9058	0.7622	0.8996	0.774
vgg16	0.8788	0.7407	0.8837	0.7317	0.8812	0.7362
vgg19	0.8864	0.75	0.8864	0.75	0.8864	0.75
Mean	0.901	0.7928	0.907	0.7805	0.9039	0.7864
Max	0.9157	0.8497	0.9363	0.8171	0.9222	0.8202
Min	0.8788	0.7407	0.8837	0.7317	0.8812	0.7362

**Table 12:** F8C AUG300 evaluation.

CNN models	Matrices					
	Precision		Recall		F1-Score	
	**class 1**	**class 2**	**class 1**	**class 2**	**class 1**	**class 2**
densenet169	0.9144	0.8742	0.9474	0.8049	0.9306	0.8381
densenet201	0.9451	0.8101	0.9058	0.8841	0.925	0.8455
resnet101	0.9218	0.8144	0.9141	0.8293	0.9179	0.8218
resnet152	0.9003	0.875	0.9501	0.7683	0.9245	0.8182
senet154	0.897	0.8077	0.9169	0.7683	0.9068	0.7875
vgg16	0.8992	0.8038	0.9141	0.7744	0.9066	0.7888
vgg19	0.8653	0.8058	0.9252	0.6829	0.8942	0.7393
Mean	0.9062	0.8273	0.9248	0.7875	0.9151	0.8056
Max	0.9451	0.875	0.9501	0.8841	0.9306	0.8455
Min	0.8653	0.8038	0.9058	0.6829	0.8942	0.7393

### Results after the experiment

Table 13 presents the results of our models after adjusting the learning rate based on the“find_lr”analysis from the fastai library. This analysis revealed the impact of different learning rates on the model’s loss function. By adjusting the learning rate from its default or fixed value, we tailored the hyperparameter to the specific behavior of each model and dataset. The results indicate that the 4C-AUG300 approach, which uses four classes with balanced augmentation and adds 300 extra images to the underrepresented classes, achieves the best overall performance. This approach not only has the highest average model score but also includes the best and worst-performing models within the group. This suggests that other approaches might benefit from more training epochs due to the increased complexity of classifying eight classes or dealing with more complex data. In the 4-class setting, the standard GAN model exhibited unstable training and difficulty in distinguishing fine-grained defect levels, which led to less diverse synthetic outputs and limited class coverage. The baseline model trained on the imbalanced dataset without augmentation achieved noticeably lower performance, confirming the need for effective oversampling techniques.

**Table 13:** Models evaluation by class recall mean after LR changes.

CNN models	Approaches			
	4 C—AUG	4C—AUG300	8 C—AUG	8C—AUG300
densenet169	**0.8903**	0.8878	0.8626	0.8797
densenet201	0.8287	0.8872	**0.89**	0.8706
resnet101	0.8118	**0.8814**	0.842	0.8614
resnet152	0.8601	**0.9013**	0.8495	0.8589
senet154	0.7972	**0.806**	0.7977	0.8019
vgg16	0.8304	0.8382	**0.8509**	0.8263
vgg19	0.8334	**0.8578**	0.808	0.8168
Mean	0.836	**0.8657**	0.843	0.8451
Max	0.8903	**0.9013**	0.89	0.8797
Min	0.7972	**0.806**	0.7977	0.8019

Tables 14, 15, 16, 17 present a detailed evaluation of each model’s precision, recall, and F1-score for both classes in each approach listed in Table 13. These results demonstrate a significant improvement in the performance of the best approach after the experiment.

**Table 14:** F4C AUG evaluation after LR changes.

CNN models	Matrices					
	Precision		Recall		F1-Score	
	**class 1**	**class 2**	**class 1**	**class 2**	**class 1**	**class 2**
densenet169	0.9398	0.8125	0.9086	0.872	0.9239	0.8412
densenet201	0.8772	0.8657	0.9501	0.7073	0.9122	0.7785
resnet101	0.8798	0.7547	0.892	0.7317	0.8858	0.743
resnet152	0.9157	0.7929	0.903	0.8171	0.9093	0.8048
senet154	0.8991	0.6346	0.7895	0.8049	0.8407	0.7097
vgg16	0.8964	0.756	0.8864	0.7744	0.8914	0.7651
vgg19	0.8898	0.8039	0.9169	0.75	0.9031	0.776
Mean	0.8997	0.7743	0.8924	0.7796	0.8952	0.774
Max	0.9398	0.8657	0.9501	0.872	0.9239	0.8412
Min	0.8772	0.6346	0.7895	0.7073	0.8407	0.7097

**Table 15:** F4C AUG300 evaluation after LR changes.

CNN models	Matrices					
	Precision		Recall		F1-Score	
	**class 1**	**class 2**	**class 1**	**class 2**	**class 1**	**class 2**
densenet169	0.9306	0.8424	0.928	0.8476	0.9293	0.845
densenet201	0.9371	0.8114	0.9086	0.8659	0.9226	0.8378
resnet101	0.9341	0.8011	0.903	0.8598	0.9183	0.8294
resnet152	0.9412	0.8512	0.9307	0.872	0.9359	0.8614
senet154	0.8854	0.7045	0.856	0.7561	0.8704	0.7294
vgg16	0.8943	0.8013	0.9141	0.7622	0.9041	0.7813
vgg19	0.9	0.869	0.9474	0.7683	0.9231	0.8155
Mean	0.9175	0.8116	0.9125	0.8188	0.9148	0.8143
Max	0.9412	0.869	0.9474	0.872	0.9359	0.8614
Min	0.8854	0.7045	0.856	0.7561	0.8704	0.7294

**Table 16:** F8C AUG evaluation after LR changes.

CNN models	Matrices					
	Precision		Recall		F1-Score	
	**class 1**	**class 2**	**class 1**	**class 2**	**class 1**	**class 2**
densenet169	0.9141	0.811	0.9141	0.811	0.9141	0.811
densenet201	0.9266	0.8726	0.9446	0.8354	0.9355	0.8536
resnet101	0.8933	0.8267	0.928	0.7561	0.9103	0.7898
resnet152	0.8984	0.8344	0.9307	0.7683	0.9143	0.8
senet154	0.8747	0.7169	0.8698	0.7256	0.8722	0.7212
vgg16	0.8987	0.84	0.9335	0.7683	0.9158	0.8025
vgg19	0.8723	0.7785	0.9086	0.7073	0.8901	0.7412
Mean	0.8969	0.8114	0.9185	0.7674	0.9075	0.7885
Max	0.9266	0.8726	0.9446	0.8354	0.9355	0.8536
Min	0.8723	0.7169	0.8698	0.7073	0.8722	0.7212

**Table 17:** F8C AUG300 evaluation after LR changes.

CNN models	Matrices					
	Precision		Recall		F1-Score	
	**class 1**	**class 2**	**class 1**	**class 2**	**class 1**	**class 2**
densenet169	0.9316	0.8046	0.9058	0.8537	0.9185	0.8284
densenet201	0.9237	0.8012	0.9058	0.8354	0.9147	0.8179
resnet101	0.9062	0.8487	0.9363	0.7866	0.921	0.8165
resnet152	0.8984	0.8865	0.9557	0.7622	0.9262	0.8197
senet154	0.8844	0.6927	0.8476	0.7561	0.8656	0.723
vgg16	0.8955	0.7427	0.8781	0.7744	0.8867	0.7582
vgg19	0.8699	0.8496	0.9446	0.689	0.9057	0.7609
Mean	0.9014	0.8037	0.9106	0.7796	0.9055	0.7892
Max	0.9316	0.8865	0.9557	0.8537	0.9262	0.8284
Min	0.8699	0.6927	0.8476	0.689	0.8656	0.723

**Fig. 6:**
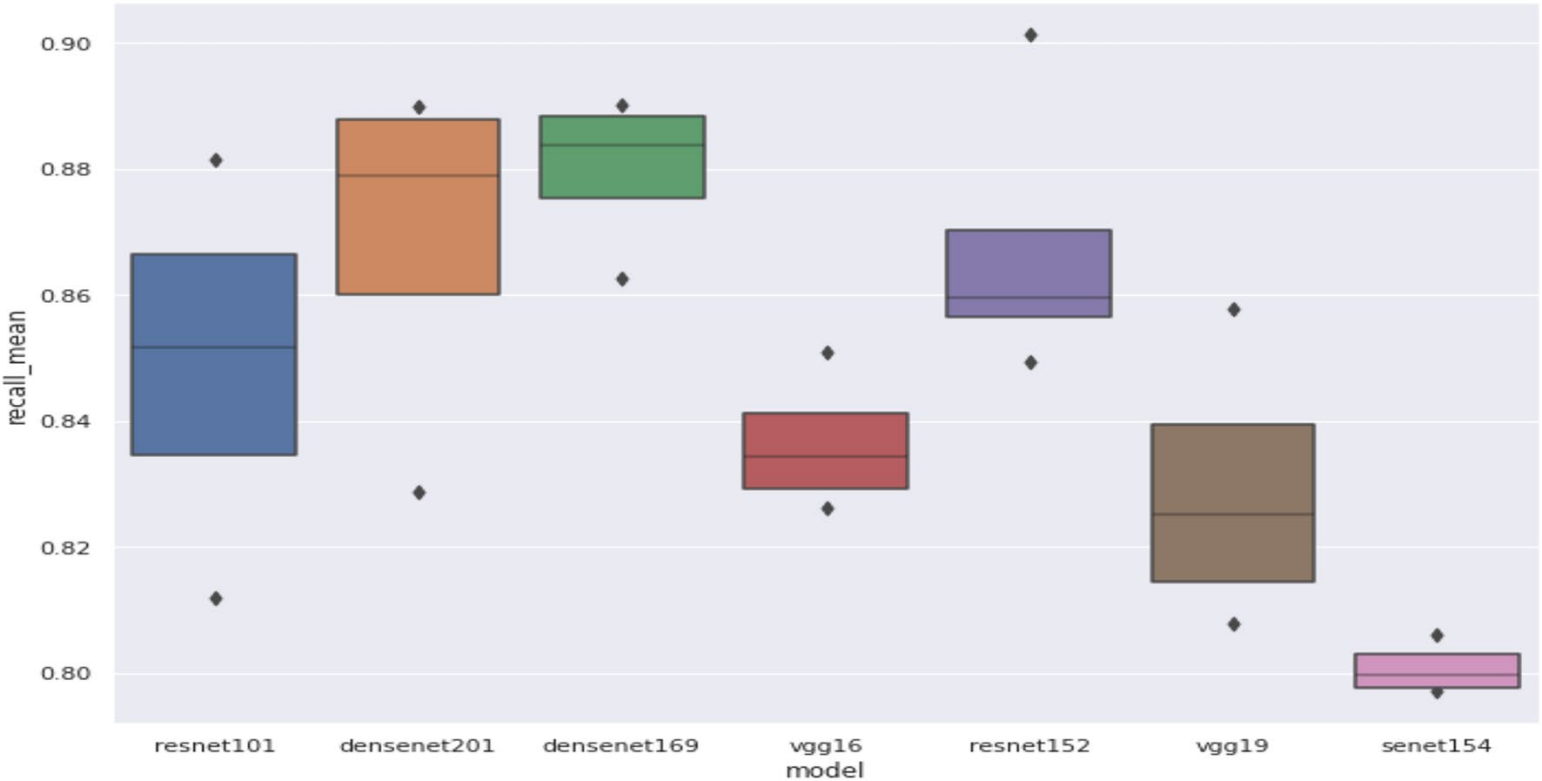
Models evaluation by class recall mean after LR changes.

Figure 6 illustrates that the ResNet152 model within the 4C_AUG300 approach consistently outperforms other models, even in the“After Experiment”stage. While DenseNet201 models generally exhibit strong performance, ResNet152 includes an outlier that stands out as the best model in this stage. This exceptional performance can be attributed to the fact that one of the ResNet152 models was trained using the optimal learning rate identified through the“find_lr”method.

**Fig. 7:**
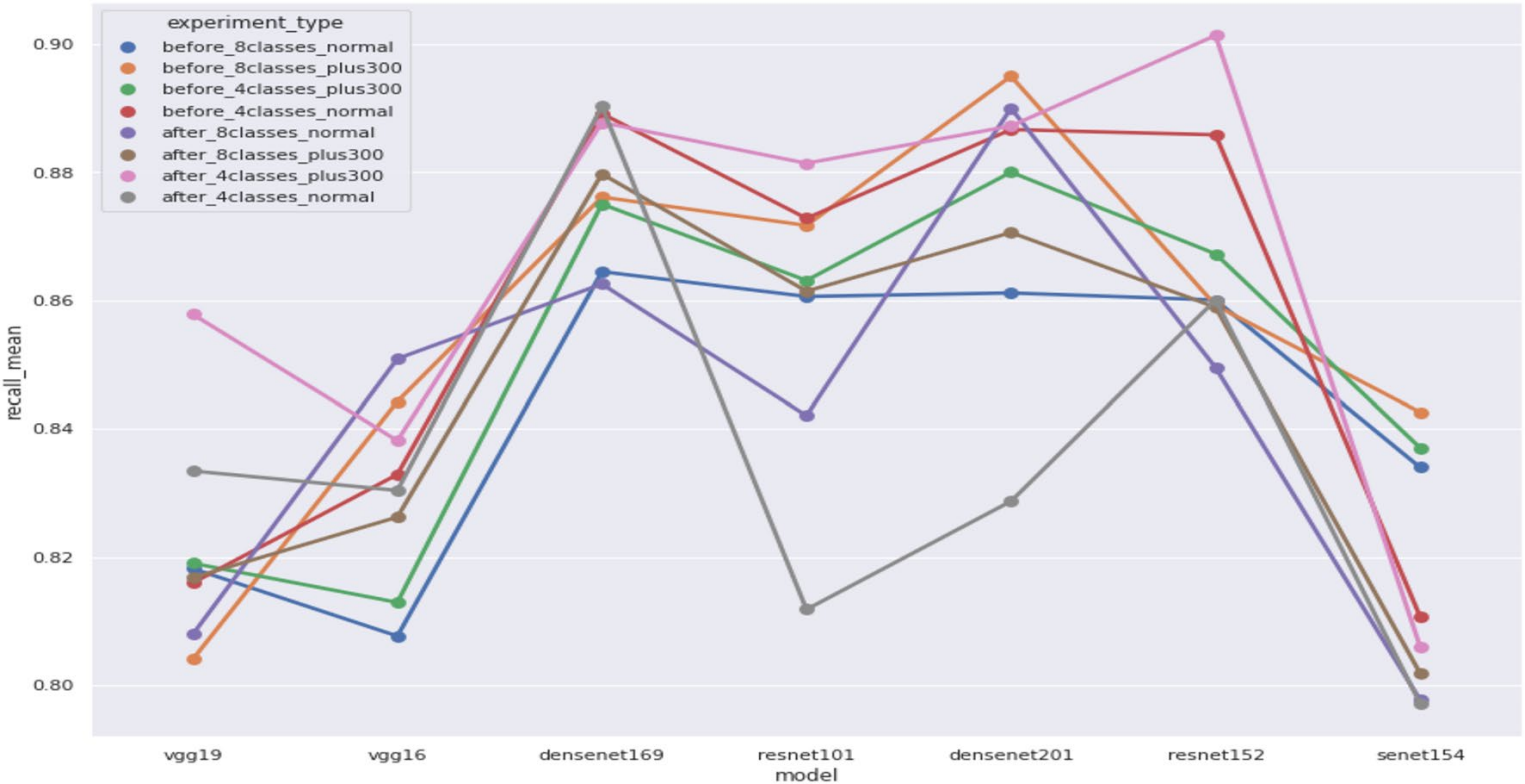
Overall models evaluation by class recall mean before and after in figureges.

Figure 7 reveals that the 4C-AUG300 approach, which uses four classes with balanced augmentation and adds 300 extra images, achieves the best overall performance, even after the experiment stage. The 4C-AUG approach, which also uses four classes but without the additional 300 images, ranks second, while the 8C-AUG300 approach, which uses eight classes with balanced augmentation and adds 300 extra images, comes in third. These results demonstrate that the“find_lr”method can be beneficial but doesn’t eliminate the need for experimentation. It simply reduces the risk of choosing suboptimal learning rates that could lead to model divergence. Table 18 presents the computational cost of each approach. The most time-consuming approach is 8C-AUG300, which uses eight classes with extensive image augmentation. This approach requires more time compared to the four-class approaches due to the increased complexity of balancing the dataset with more classes. Despite its higher computational cost, the DenseNet201 model within the 8C-AUG300 approach still achieves a reasonable training time of 134 epochs. This suggests that the trade-off between accuracy and computational cost is acceptable for real-world implementation. The ResNet152 model within the 4C-AUG300 approach offers the best balance of accuracy and computational efficiency. It achieves the highest overall accuracy among all approaches while maintaining a relatively low training time of 116 epochs. This makes it an excellent choice for real-world applications where both accuracy and efficiency are crucial.

**Table 18:** Models evaluation average time per epochs.

CNN models	Approaches	
	4C—AUG300	8C—AUG300
	Time (seconds)	Time (seconds)
densenet169	70	96
densenet201	101	134
resnet101	91	124
resnet152	116	157
senet154	225	308
vgg16	**64**	**88**
vgg19	96	129

**Fig. 8:**
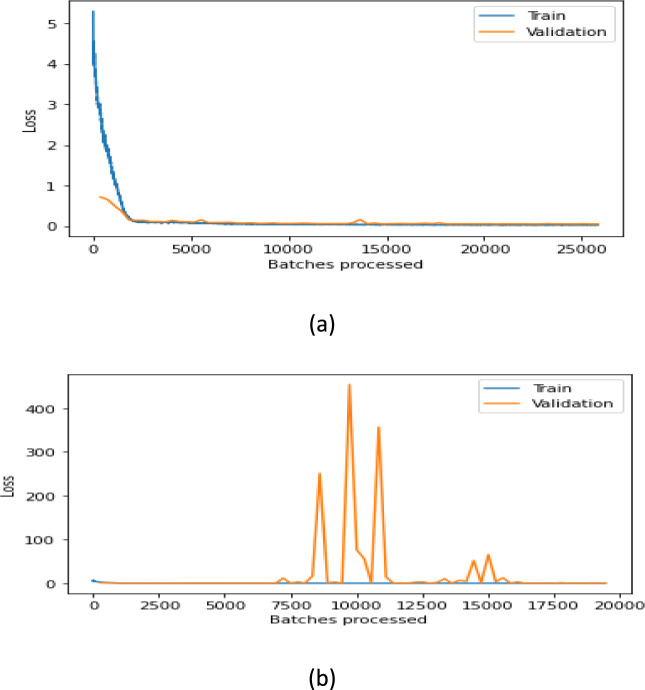
Relation between batch process and loss for train and validation by class recall.

Figure 8 compares the performance of the best two models, models (a) and (b), across all approaches, both before and after the experiment. While both models exhibit similar trends, model (b) demonstrates an unusual pattern in its loss function. This behavior can be attributed to the adaptive learning rate adjustment based on the“find_lr”method. The“find_lr”method can sometimes suggest relatively high learning rates. When combined with the cyclic learning rate scheduler used in fastai, these high learning rates can lead to temporary increases in loss during certain phases of the training cycle. This is likely the cause of the peaks observed in model (b)’s loss curve. As the learning rate decreases during subsequent cycles, the loss function returns to a more normal level.

## Conclusions

DenseNet and ResNet consistently emerge as top-performing models across our approaches, aligning with their established reputation in various research domains. Our findings demonstrate that GAN-based oversampling techniques, such as the 4C-AUG300 and 8C-AUG300 approaches, significantly enhance the performance of these models. While ResNet152 and DenseNet201 variants exhibit exceptional results, deeper models often require more computational resources and time. By employing GAN-based oversampling, we not only identified the best two models but also achieved the best average performance across all approaches, except for the normal GAN with four classes in the pre-experiment stage. The difference between the 8C-AUG300 and 4C-AUG approaches is minimal (0.0002), highlighting the effectiveness of oversampling small classes in improving both individual model performance and overall approach effectiveness. Furthermore, our results emphasize the importance of carefully selecting the threshold value for classification. Blindly adopting a threshold of 0.5 can lead to suboptimal performance. By analyzing model behavior on validation data, we can automatically determine a more suitable threshold, eliminating the need for manual intervention and improving overall accuracy. While this study primarily focused on enhancing classification accuracy and robustness, we recognize the importance of inference time efficiency for real world deployment. Future work will include a detailed analysis of the tradeoff between speed and accuracy, as well as the exploration of lightweight alternatives and optimization techniques such as pruning and quantization to support deployment on resource constrained systems. Additionally, the proposed methodology demonstrates potential for generalization to other anomaly detection tasks that involve complex image data with class imbalance and subtle defect patterns. Domains such as industrial inspection, electronic component analysis, and medical imaging may particularly benefit from adapting this framework. Future work will explore its effectiveness and adaptability in such applications. Despite the promising results, this study has certain limitations. The reliance on EL image datasets means that performance may vary when applied to different imaging modalities or real-time operational environments. Additionally, the computational cost of training deep models like ResNet152 and DenseNet201 may pose challenges for deployment on low-resource devices. From a practical standpoint, the proposed framework can assist in the early and automated identification of PV cell defects, potentially improving inspection speed and reducing labor costs in photovoltaic manufacturing and maintenance. Future research will focus on validating the framework across diverse datasets, integrating real-time inference mechanisms, and exploring model compression strategies to improve deployment feasibility in edge computing environments.

## Data Availability

All data generated or analyzed during this study are included in this published article. You can contact M. A. Ebied in case of requesting study data. this email: dr.m_ebied@techedu.bsu.edu.eg.
